# Discovery of cationic nonribosomal peptides as Gram-negative antibiotics through global genome mining

**DOI:** 10.1038/s41467-018-05781-6

**Published:** 2018-08-16

**Authors:** Yong-Xin Li, Zheng Zhong, Wei-Peng Zhang, Pei-Yuan Qian

**Affiliations:** 1 Department of Ocean Science and Division of Life Science, Hong Kong University of Science and Technology, Clear Water Bay, Hong Kong China; 2 Institute for Advanced Study, Hong Kong University of Science and Technology, Clear Water Bay, Hong Kong China

## Abstract

The worldwide prevalence of infections caused by antibiotic-resistant Gram-negative bacteria poses a serious threat to public health due to the limited therapeutic alternatives. Cationic peptides represent a large family of antibiotics and have attracted interest due to their diverse chemical structures and potential for combating drug-resistant Gram-negative pathogens. Here, we analyze 7395 bacterial genomes to investigate their capacity for biosynthesis of cationic nonribosomal peptides with activity against Gram-negative bacteria. Applying this approach, we identify two novel compounds (brevicidine and laterocidine) showing bactericidal activities against antibiotic-resistant Gram-negative pathogens, such as *Pseudomonas aeruginosa* and colistin-resistant *Escherichia coli*, and an apparently low risk of resistance. The two peptides show efficacy against *E. coli* in a mouse thigh infection model. These findings may contribute to the discovery and development of Gram-negative antibiotics.

## Abstract (web summary)

Microbial genomes encode enzymes for biosynthesis of many uncharacterized peptides. Here, the authors screen over 7,300 bacterial genomes for potential biosynthesis of cationic non-ribosomal peptides, and identify two novel peptides with activities against antibiotic-resistant Gram-negative pathogens.

## Introduction

Infection due to multidrug-resistant Gram-negative bacteria has become a major threat to public health^1,2^. The worldwide search for a truly novel class of antibiotics that can combat such bacteria and overcome antibiotic resistance has continued for the last 40 years^1–4^. Cationic peptides are found in all forms of life and are among the most widespread and structurally diverse antibiotics in nature. Carrying unique chemical properties that enable them to not only penetrate those outer membranes that are highly impermeable, but also interact with multiple anionic intracellular targets, cationic peptides are highly effective against drug-resistant Gram-negative pathogens^5–9^. In the past four decades, thousands of cationic peptides with broad antimicrobial activities have been identified with most of them being either natural or naturally-derived host–defense peptides from multicellular organisms. However, only a handful of these cationic peptides have entered clinical application, due to their high cost of supply, proteolytic instability, and unknown toxicological profile^8–14^.

Global analysis of sequenced bacterial genomes indicates that nonribosomal peptides (NRPs) are among the most widespread and structurally diverse families of complex secondary metabolites, the vast majority of which encode unknown natural products^15–17^. Among these NRPs, cationic nonribosomal peptides (CNRPs) with their sufficient supply and proteolytic stability are attractive therapeutic candidates to combat Gram-negative pathogens. Polymyxins and gramicidin S produced by Bacilli bacteria are in fact among the few precedents with clinical efficacy^12–14^. However, the diversity and complexity of CNRPs have made systematic investigation difficult and consequently, the vast majority of genetically encoded CNRPs in bacteria have been overlooked.

**Fig. 1:**
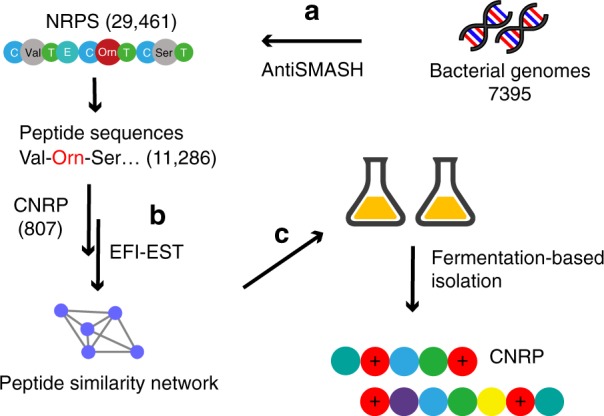
Workflow of the global genome mining of CNRP BGCs. **a** 7395 complete or draft bacterial genomes were downloaded from GenBank and subjected to stand-alone antiSMASH for BGC analysis and NRP sequence prediction, leading to the identification of 29,461 NRPS BGCs, in which 11,286 BGCs encoded NRPs with positively charged residues. **b** Selected peptide sequences of cationic peptides (numbering 807) were submitted to the EFI–Enzyme Similarity Tool (EFI–EST) for constructing the peptide similarity network. **c** The CNRPs with desirable chemical features were obtained by conventional fermentation-based isolation

In the present study, we adopt a global genome-mining approach to provide insight into the biosynthetic capacity of CNRPs and facilitate the genome-guided discovery of antibiotics (Fig. 1). By screening 7395 bacterial genomes for the potential biosynthesis of cationic nonribosomal peptides, we identify two novel peptides with activities against antibiotic-resistant Gram-negative pathogens.

## Results

### Global analysis of genetically encoded CNRPs

**Fig. 2:**
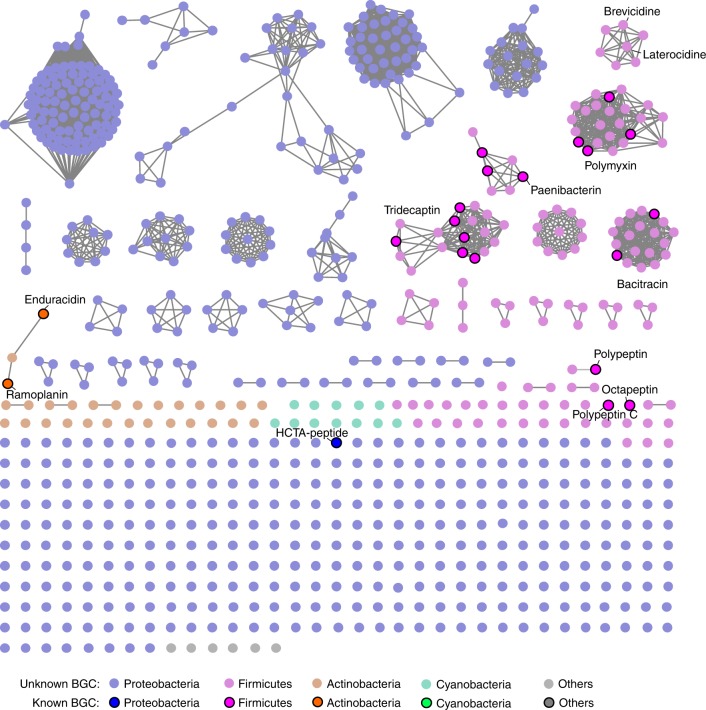
A peptide similarity network of the CNRPs showing their diversity, distribution, and discovery status. Clearly, the majority of CNRPs remain unexplored, particularly the ones in the Firmicutes and Proteobacteria phyla

Current genome-mining strategies rely heavily on comparing sequences with known biosynthetic genes or domains to identify orphan biosynthetic gene clusters (BGCs)^17–19^. Here, we developed a genome-mining approach based on antiSMASH^19^ that effectively targets NRP BGCs by parsing the chemical features of predicted NRPs rather than the sets of domains or protein sequences that are phylogenetically closely related (Fig. 1). To gain insight into the diversity of bacterial CNRPs, we bioinformatically analyzed 5585 complete bacterial genomes for BGCs encoding CNRPs. In total, 665 out of 6879 genetically encoded NRPs contained two or more positively charged amino acids (i.e., arginine, histidine, lysine, ornithine, and 2,4-diaminobutyric acid), which were mainly distributed in Proteobacteria, Actinobacteria, and Firmicutes (Supplementary Fig. 1). Further, draft genomes (numbering 1810) from the top 20 genera in terms of CNRP abundance as well as 5585 complete genomes were chosen for global genome mining of CNRPs. A total of 11,286 BGCs encoding NRPs with positively charged residues were detected from 7395 bacterial genomes. Of the 11,286 NRPs, 2801 were putative CNRPs containing two or more positively charged residues. This number is four times that generated from data sets of the complete bacterial genomes. Further, BGC analysis of these putative CNRPs excluding peptides with *N*-formyl-N-OH-Lys/Orn residues (i.e., siderophores)^20^ or shorter peptides led to the identification of 807 CNRPs (≥6 residues and ≥2 positively charged residues) that are extremely diverse and widely distributed in bacteria (Fig. 2). Most of these CNRPs (>95%) varied from 6 to 18 in length with an average of 9.8 (median 9), while their percentage of positively charged residues ranged from 6.3 to 77.8% with an average of 30.6% (median 28.6%) (Supplementary Fig. 2).

To obtain insight into the structural diversity and novelty of CNRPs, we next applied the EFI–Enzyme Similarity Tool (EFI–EST)^21^ to directly chart the chemical space of genetically encoded CNRPs using the predicted peptide sequence of chemical compounds rather than the sequence of proteins or domains as in previous methods^18,22,23^. A sequence similarity network^21^ consisting of 807 CNRPs as well as 24 known CNRPs was then constructed to visualize the diversity, distribution, and discovery status of CNRPs (Fig. 2). It immediately became clear that the majority of CNRPs were unexplored, with most of them mainly harbored by Proteobacteria and Firmicutes. Our analysis suggests that 793 out of the 807 predicted CNRPs did not have any isolated members. In particular, 738 could not be grouped into the known classes of CNRPs, reiterating our finding that the vast majority of genetically encoded CNRPs have yet to be discovered. The volume of bacterial genomic data is increasing by leaps and bounds every day. Our approach not only offers insight into the biosynthetic capacity of NRPs, but also enables identifying and prioritizing novel BGCs of interest within large data sets in a robust manner for the genomics-based discovery of natural products.

### Leveraging structure prediction for CNRP discovery

We next sought to leverage network analysis-associated prioritization to facilitate the targeted, genome-guided discovery of novel CNRPs. *N*-acylated CNRPs, also known as cationic lipopeptides including polymyxins and tridecaptins^24^, represent a growing family of attractive candidate antibiotics as they have shown high efficacy against infections caused by Gram-negative pathogens. Our global analysis suggests that 261 CNRPs (or 32.3%) were putative cationic lipopeptides. Intriguingly, 254 (or 97.3%) of them did not have any isolated members. In particular, 221 (or 84.7%) could not be grouped into the known classes of CNRPs (Supplementary Figs. 3–4). These CNRPs therefore likely represent novel classes of cationic lipopeptides. Among these diverse BGCs for cationic lipopeptides, 116 falling into 12 classes were harbored by Bacilli. Since cryptic metabolites have proven to be a major bottleneck in genome-guided discovery, we selected Bacilli for the fermentation-based discovery of antibiotics as this class of bacteria are well known for producing CNRP antibiotics^24^. Traditional fermentation-based isolation was then performed focusing on putative cationic lipopeptides with three or more positively charged residues, as more positively charged residues enable electrostatic interaction with negatively charged cell membranes while the hydrophobic acyl chain defines membranolytic and cell-penetrating properties^7^.

**Fig. 3:**
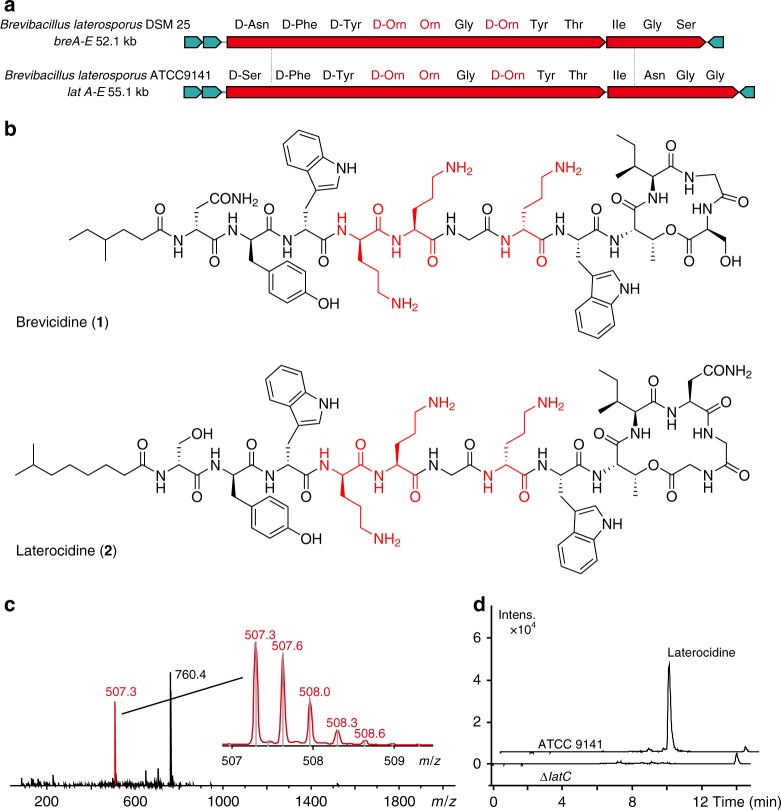
Cationic antibacterial peptides discovered via global genome mining. **a** Two gene clusters of investigated cationic peptides, with amino acids incorporated. Cationic residues are marked in red. **b** Structure of isolated cationic peptides identified by MS/NMR analysis. Positively charged amino acids are highlighted in red. **c** Mass spectra (ESI) of cationic peptide brevicidine. **d** Liquid chromatography-mass spectrometry traces comparing *B. laterosporus* ATCC 9141 wild type and the Δ*latC* mutant. Figure 3 **c**–**d** are representative of three independent experiments

*Brevibacillus laterosporus* DSM 25, ATCC 9141, and *Paenibacillus alvei* DSM 29 of the Bacilli that harbored active BGCs of interest on the basis of genome mining and metabolic analysis were selected for further study (Methods and Fig. 3a–b). Metabolic analysis of selected strains using UPLC-MS-ESI showed a typical ion pattern of cationic peptides (i.e., MW > 1000 and an abundance of doubly/triply charged ions) (Fig. 3c). Cationic lipopeptides (**1**–**3**) and their derivatives were subsequently isolated from three strain cultures and characterized structurally by MS/MS, NMR, and Marfey-type analyses (Fig. 3b and Supplementary Figs. 5–7). Compound **1** was obtained as a white amorphous solid from strain DSM 25. On the basis of HRESIMS data (*m/z* 760.4046 [M + 2 H]^2+^, calculated 760.4064), we established its molecular formula to be C_74_H_106_N_18_O_17_. The characteristic signals of the amide proton in the ^1^H-NMR spectrum and of carbonyl groups in the ^13^C NMR spectrum (Supplementary Notes) indicated the peptidic nature of compound **1**. The gross structure of **1** was further established by analyses of the ^1^H, ^13^C, ^1^H–^1^H COSY, HMQC, and HMBC NMR spectral data (Supplementary Fig. 5b), revealing the presence of Orn (3), Trp (2), Gly (2), Ile (1), Ser (1), Thr (1), Asn(1), Tyr (1), and a lipidic residue that was identified as 4-methylhexanoic acid. The gross structure established by NMR analysis was also corroborated by MS/MS analysis (Supplementary Fig. 5a). On the basis of advanced Marfey’s analysis for the absolute configuration of amino acids (Supplementary Fig. 5c), a lipodepsipeptide 4-Methyl-Hexanoyl-D-Asn-D-Tyr-D-Trp-D-Orn-Orn-Gly-D-Orn-Trp-Thr-Ile-Gly-Ser (cyclized via lactone formation between Thr and the C terminus) was established for compound **1**.

These newly identified CNRPs, named brevicidine (**1**), laterocidine (**2**), and paenibacterin B (**3**), are new cyclic depsipeptides with an *N*-acylated side chain and a lactone ring that formed through the condensation of a C-terminal residue with a threonine (Thr) residue. Paenibacterin B is a new derivative of paenibacterin (Supplementary Fig. 7), which is a bactericidal antibiotic binding to the bacterial outer membrane^25^. Together with well-known peptide antibiotics including polymyxins, ramoplanin, and enduracidin, these CNRPs belong to a large family of cyclic cationic lipopeptides that are acylated at the N terminus by a lipid tail and cyclized between the C terminus and the internal residue with an amine or hydroxyl group via an amide or ester bond. Brevicidine and laterocidine form a new class of cyclic cationic peptides exhibiting two unique structural features: a linear cationic segment with three positively charged ornithine residues, and a small hydrophobic tetrapeptide/pentapeptide ring (Fig. 3b). They are different from previously reported cyclic cationic lipopeptides that share the structural feature of a positively charged macro-ring (Supplementary Fig. 8).

Predicted biosynthesis of laterocidine started with a typical starting module for *N*-acylated NRPs with a condensation starter domain that acylated the first amino acid with a fatty acid. In accordance with the co-linearity rule, the other 13 amino acids, including positively charged residues (D-Orn_4,_ L-Orn_5_, and D-Orn_7_), were introduced into the linear peptide and the ring closure between the last Gly_13_ and Thr_9_ was catalyzed by a thioesterase domain during product offloading (Supplementary Fig. 9). A similar biosynthetic assembly line was also adopted by brevicidine biosynthesis (Supplementary Fig. 10). To directly link the predicted CNRP BGCs to their isolated putative CNRPs, we applied the CRISPR/Cas9 gene editing system^26–28^ to generate a Δ*latC* mutant strain of *Brevibacillus laterosporus* ATCC 9141 for metabolic analysis. The complete abolishment of laterocidine production in the Δ*latC* mutant in UPLC-MS analysis (Fig. 3d) suggests gene cluster *lat*A-E to be the BGC of laterocidine, thus directly linking the CNRPs predicted from global genome mining to the isolated natural product. Altogether, our results demonstrate the power of global genome mining for the targeted, genome-guided discovery of novel CNRPs.

### Antibiotic activity, resistance, and mechanism

**Fig. 4:**
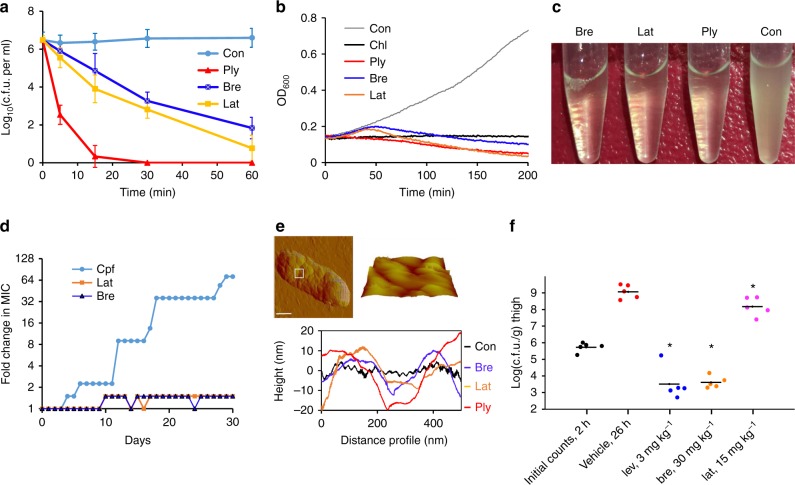
Brevicidine and laterocidine show bactericidal activity, a low risk of resistance and efficacy in a mouse thigh infection model. **a** Time-kill assays. *E. coli* were grown to early phase and challenged with ten times the MIC of antibiotics. Data are representative of three independent experiments ± s.d. **b** Bacterial growth kinetics. Optical densities of *E. coli* cells exposed to two times the MIC of antibiotics. **c** CNRP treatment resulted in the lysis of *E. coli*. Figures **b**, **c** are representative of three independent experiments. **d** Resistance acquisition during serial passaging in the presence of sub-MIC levels of antimicrobials. **e** Atomic force microscopy of *E. coli* grown at 37 °C to mid-log phase (OD_600_ of ∼0.5) in the presence of ten times the MIC of CNRP antibiotics. Scale bar is 1 µm. The white square highlights the region scanned to obtain high-resolution topographical images of the brevicidine-treated cell surface. Scans are representative of two independent experiments. **f** Efficacy of brevicidine and laterocidine in a mouse thigh model infected by *E. coli*. Brevicidine (30 mg kg^−1^) or laterocidine (15 mg kg^−1^) treatment (twice) of *E. coli* thigh infections (five mice) leads to a reduced number of viable bacteria after 26 h. Significant differences between groups analyzed by the Mann–Whitney test (*P < 0.01). For **a**–**f**, con negative control, bre brevicidine, lat laterocidine, ply polymyxin, lev levofloxacin, chl chloramphenicol, and cfp ciprofloxacin

All of the isolated compounds **1**–**11** (Supplementary Fig. 11) were assessed for bioactivities against ESKAPE bacteria (*Enterococcus faecium*, *Staphylococcus aureus*, *Klebsiella pneumoniae*, *Acinetobacter baumannii*, *Pseudomonas aeruginosa*, and *Enterobacter* species), which cause the majority of hospital-acquired infections^2^. Both brevicidine and laterocidine exhibited broad antibacterial activities against Gram-negative bacteria including opportunistic pathogens such as difficult-to-treat *P. aeruginosa* and colistin-resistant *E. coli*. Minimal inhibitory concentration (MIC) values in the micromolar range (1–16 μg mL^−1^; 0.66–10.5 μM) testified to their high potency. Further, the antibacterial activities of compounds **1**–**2** were not impaired in the presence of fetal bovine serum (Table 1). Harboring unnatural amino acids such as d-amino acids, a fatty acid side chain and a lactone ring, the two cyclic lipopeptides were proteolytically stable. Our preliminary structure–activity relationship study suggests that both the linear cationic segment and the hydrophobic lactone ring were crucial for their activities. For example, the lack of either the lactone ring or the cationic side chain dramatically increased MIC values 8- to 32-fold (**1** vs. **4–5**, **2** vs. **9–11**) (Supplementary Table 1). The two CNRPs **1** and **2** were bactericidal against *E. coli* as revealed by the growth kinetics of *E. coli*, time-kill assay, and lysis assay (Fig. 4a–c). Both the time-kill assay and growth kinetics showed time-dependent reductions in the number of colony-forming units per mL (c.f.u. mL^−1^) or in the OD_600_ value for *E. coli* treated by compounds **1**–**2**. The *E. coli* cells were completely lysed at ten times the MIC, similar to polymyxin B. Compounds **1**–**2** neither caused the lysis of erythrocytes nor showed significant toxicity against the human cell line HeLa at concentrations up to 128 μg mL^−1^ (Supplementary Fig. 12), suggesting that they are promising candidates for Gram-negative antibiotics.

**Table 1:** Activity of CNRPs against microorganisms

Target species	MIC (µg mL^−1^)			
	**1**	**2**	Polymyxin B	Colistin
*Escherichia coli* ATCC 25922	2	2	1	2
Colistin-resistant *E. coli* ^a^	2	2	16	16
*E. coli* ATCC 25922 + 10% serum	2–4	2–4	1–2	2–4
*E. coli* ATCC 25922 + Mg^2+^ (21 mM)	>64	>64	>64	>64
*E. coli* ATCC 25922 + LPS (1.0 mg mL^−1^)	>64	>64	>64	>64
*E. coli* TOP10	2	2	2	2
*Pseudomonas aeruginosa* PAO1	1	2	1	1
*Acinetobacter baumannii*	16	4	4	
*Klebsiella pneumoniae* NRRL-B-408	2	4	16	
*K. pneumoniae* NRRL-B-3521	4	4	2	
*Enterobacter cloacae* NRRL-B-413	2	2	8	
*E. cloacae* NRRL-B-425	2	2	64	
*Bacillus subtilis* 168	32	32	4	
*Candida albicans*	>64	>64	>64	
*Saccharomyces cerevisiae*	>64	>64	>64	
*Staphylococcus aureus* (MRSA)	>64	>64	32	

^a^*E. coli* ATCC 25922 carrying *mcr-1*

To explore the mode of action of compounds **1**–**2**, we initially attempted to identify resistant mutants of *E. coli* ATCC 25922. Interestingly, *E. coli* did not develop resistance during continuous serial passaging in the presence of subinhibitory concentrations of either **1** or **2** over a 30-day period (Fig. 4d), suggesting a low risk of resistance to compounds **1**–**2**. By contrast, *E. coli* developed resistance to ciprofloxacin within only a few days of exposure (Fig. 4d). *E. coli* did not develop resistance to **1** either when plated on media at four times the MIC. Neither **1** nor **2** exhibited antibiotic effects when cells were grown in the presence of exogenous lipopolysaccharides (LPS) or a high concentration of Mg^2+^ as expected (Table 1), suggesting that these cationic peptides disrupted the integrity of the outer membrane through association with LPS. This suggestion was further supported by atomic force microscopy (AFM) and isothermal titration calorimetry analysis. AFM analysis of *E. coli* ATCC 25922 and *P. aeruginosa* PAO1 treated with compound **1** or **2** showed that treated cells lost their regular rod-like shape and became lumpy and rugged (Fig. 4e and Supplementary Figs. 13–14). The morphology of compound-treated cells was extensively disrupted as characterized by undulations on the order of ~30 nm in amplitude, whereas untreated *E. coli* remained uniform. Indeed, compound **1** displayed high affinity for LPS in vitro (dissociation constant *K*_d_ of 6.5 µM), similar to polymyxin B (Supplementary Fig. 15). Notably, the antibacterial activities of both brevicidine and laterocidine were retained against colistin-resistant *E. coli* ATCC 25922 carrying *mcr-1* (Table 1), suggesting that the action of the two CNRPs is independent of phosphoethanolamine modifications in lipid A^29^. Collectively, the data suggest that compounds **1**–**2** represent an attractive therapeutic alternative for combating drug-resistant Gram-negative bacteria expressing *mcr-1*, which poses a significant threat to public health worldwide.

The growth kinetics of *E. coli* cells showed that cells exposed to compound **1** or **2** grew at a reduced rate for 30 min, after which a steady decrease in cell count was observed (Fig. 4a). These data are different from those of polymyxins, which are generally bactericidal within minutes of exposure due to the formation of large, nonspecific pores in the bacterial membrane^30,31^. In the time-kill assays, *E. coli* cells showed slightly reduced cell viability after 15 min of exposure and a significant reduction after 30 min of exposure to **1** or **2** at ten times the MIC. Polymyxin B acted more quickly, significantly reducing the viable cell population after 5 min and killing all cells within 30 min. These results suggest that compounds **1**–**2** may be membrane-targeting bactericidal peptides but may not act through a generic membrane lysis mechanism-like polymyxin B does.

Studying the mode of action of cationic peptides is challenging because they have several modes of action, such that they are able to interact with multiple anionic intracellular targets. Take the well-studied cationic peptide polymyxins, for example, it is still unclear how they interact with and disrupt the cytoplasmic membrane and intracellular targets^31,32^. Although information on the mode of action of **1**–**2** is scant, the fact that no significant membrane disruption effect was observed at twice the MIC suggests that the two CNRPs could potentially have multiple targets. However, more in-depth mechanism study is needed to characterize their intracellular targets as well as their membrane disruption effect.

Given the low risk of resistance and the attractive mode of action of **1**–**2**, an animal efficacy study was then performed in a mouse thigh model. Mice were intramuscularly infected with 1.50 × 10^6^ c.f.u. of *E. coli* ATCC 25922. Two hours post infection, brevicidine (30 mg kg^−1^) or laterocidine (15 mg kg^−1^) was subcutaneously injected at two doses. Levofloxacin (MIC = 0.032 µg mL^−1^) was dosed at 3.0 mg kg^−1^ as a positive control. The two compounds showed significant antibacterial efficacy against *E. coli* in the neutropenic mouse thigh infection model. In particular, compound **1** was highly efficacious in mice infected with *E. coli* and caused a 5 log_10_ reduction in c.f.u. in the thigh compared to the vehicle control after 24 h (Fig. 4f).

## Discussion

The chemical diversity of natural cationic peptides has provided privileged scaffolds for the development of antibiotics to combat Gram-negative pathogens. These valuable metabolites are typically identified on a case-by-case basis, which is both tedious and downright inefficient. Bioinformatics methods have recently been developed to prioritize and deduplicate the genome-based discovery of natural products from large data sets^17–19,22^. However, the structural and biosynthetic diversity of genetically encoded cationic peptides has rendered their comprehensive investigation and targeted discovery extremely challenging. Here, we systematically profiled the scaffolds of bacterial NRP-derived cationic peptides, by employing a peptide sequence similarity network to sort and target these cationic peptides on the basis of their chemical features. Using chemical building blocks of NRPs predicted by well-established algorithms, our network-associated genome-mining approach provided us a rare chance to directly and rapidly examine the structures and functions of CNRPs. Our analysis not only revealed the chemical diversity of CNRPs in bacteria, but also prioritized a novel scaffold of interest for antibiotic discovery.

The increasing incidence of infection caused by multidrug-resistant Gram-negative pathogens, especially colistin-resistant pathogens, threatens to overwhelm healthcare systems worldwide. Bacteria harboring the colistin-resistant gene *mcr-1* have been found across the globe since their first discovery in 2015^29,33^. These bacteria threaten to remove the antibiotics of last resort from an already shrinking antibiotic arsenal. New antibacterial candidates with a low risk of resistance are urgently needed as conventional antibiotics are quickly becoming ineffective as a result of resistance. The present study highlights the utility of global genome mining in the systematic investigation of bacterial CNRPs and provides a tool for the targeted discovery of CNRP antibiotics. Making use of this tool and its structural prediction and prioritization capabilities, we identified two new classes of cationic antibiotics. The successful demonstration of the antimicrobial efficacy of two CNRPs against Gram-negative pathogens, especially colistin-resistant pathogens, undoubtedly distinguishes bacterial CNRPs as a new source of Gram-negative antibiotics. However, like other cationic peptides, the mode of action and toxicity of these two CNRPs must be studied in greater detail to consolidate their status as promising preclinical antibiotic candidates. Our identification of antibiotics against Gram-negative bacteria through the global genome mining of CNRPs validates the power of this strategy in discovering suitable drug candidates to combat Gram-negative pathogens that have developed antibiotic resistance. It is likely that many other cationic peptide antibiotics with high efficacy and low resistance risk are present in bacteria and are just waiting to be discovered.

## Methods

### Global genome mining of CNRPs

A total of 5585 complete bacterial genomes (with cutoff on October 20, 2016) were retrieved from NCBI Genome and subjected to BGC analysis utilizing the stand-alone version of antiSMASH v3.0.5^34^. In total, 6879 NRP BGCs were identified from complete bacterial genomes spanning the entire domain of bacteria. To gain insight into the biosynthetic capacity of bacterial CNRPs, we created a series of scripts to collect BGC information from the antiSMASH-annotated files. Costume Node.js scripts were prepared to extract predicted amino acid building blocks from adenylation (A) domains in nonribosomal peptide synthetase (NRPS). Amino acids predicted by Stachelhaus code were collected, and the frequency of occurrence in each NRPS gene cluster was determined. After analyzing all complete genomes, the genera were ranked based on their abundance in CNRP BGCs containing two or more positively charged amino acids (i.e., arginine, histidine, lysine, ornithine, and 2,4-diaminobutyric acid). Draft genomes (numbering 1810) from the top 20 genera as well as all complete genomes were chosen for global genome mining of CNRPs. The top 20 genera were *Actinoplanes, Alcanivorax, Arthrobacter, Azotobacter, Bacillus, Brevibacillus, Corallococcus, Cyanothece, Kibdelosporangium, Myxococcus, Nitrosomonas, Paenibacillus, Pandoraea, Photorhabdus, Polyangium, Pseudomonas, Saccharothrix, Sorangium, Vibrio*, and *Xenorhabdus*. Peptide sequence similarity networking was performed using the EFI–EST^21^. In total, 7395 bacterial genomes were subjected to BGC analysis, leading to the identification of 29,461 NRP BGCs. Gene clusters containing protein SMCOG1080 (lysine/ornithine *N*-monooxygenase) were excluded from this study because their encoding products were mainly NRPS-dependent siderophores with *N*-formyl-N-OH-Lys/Orn residues^20^. Costume Node.js scripts were developed to interrogate the predicted peptide sequences and convert the amino acids into their abbreviated forms. Non-proteinogenic amino acids were assigned to their proteinogenic counterparts based on their structural similarity or biosynthesis origin (Supplementary Table 2). The peptide sequences of all gene clusters were filtered according to the following rules: number of positively charged amino acids (K, H, and R) ≥ 2 and sequence length ≥6. In total, 807 peptide sequences were selected and analyzed by EFI–EST (*E* value = 10E−3, Fraction = 1). The resulting full network was visualized by Cytoscape^35^. In particular, the polymyxin family contains an extremely high proportion of Dab and a limited amino acid composition, which can lead to an inaccurate *E* value in EFI–EST. Thus, the polymyxin family (23 BGCs) was manually selected and analyzed separately using BLASTP multiple alignment to construct the network (*E* value < 10E−3).

### Metabolic analysis by UPLC-MS

On the basis of global genome mining targeting those *N*-acylated CNRPs with three or more positively charged residues, our follow-up screening focused on CNRPs in the Bacilli class which harbors the BGC group of polymyxins, tridecaptins, paenibacterins, or a putative new CNRP class (Supplementary Fig. 4). Six strains available in the bacterial collection center were selected for further metabolic analysis. *B. laterosporus* DSM 25 and ATCC 9141 harbor novel BGCs for a putative new class of CNRPs. *P. polymyxa* CICC 10580 and ATCC 842 harbor uncharacterized tridecaptin gene clusters, *P. alvei* DSM 29 harbors uncharacterized paenibacterin BGCs, and *P. assamensis* DSM 18201 harbors uncharacterized polymyxin BGCs. All of them could potentially produce new analogs of known peptide antibiotics on the basis of amino acid variation.

The six selected strains were cultivated in 5 mL of a modified Tryptic soy broth medium (MTSB, 30 g L^−1^ Tryptic Soy Broth (Sigma), 20.0 g L^−1^ starch 2.0 g L^−1^ MgSO_4_·7H_2_O, 10.0 g L^−1^ CaCO_3_) and then incubated at 30 or 37 °C on a shaker (250 rpm). After incubation for 12–48 h, each culture supernatant was extracted with Diaion® HP-20 (0.25 g; Sigma-Aldrich) and washed with 20% isopropyl alcohol (IPA) (4.0 mL) and then eluted with 80% IPA supplemented with 0.1% trifluoroacetic acid (0.50 mL). The collected partition was dried and redissolved in 100 μL of MeOH for metabolic analysis. The metabolic analysis of all samples dissolved in 100 µL of MeOH was conducted on an ultra-high-performance liquid chromatography (UPLC) system (Waters ACQUITY, with a Waters BEH C18 reversed-phase UPLC column) coupled with a Bruker microTOF-q II mass spectrometer (Bruker Daltonics GmbH, Bremen, Germany). UPLC-MS analysis of the selected strains led to the identification of *B. laterosporus* DSM 25, ATCC 9141, and *P. alvei* DSM 29, which produced putative cationic peptides with the typical ion pattern (i.e., MW > 1000 and abundant doubly/triply charged ions).

### Isolation of compounds

*B. laterosporus* DSM 25 was cultivated for 1 day at 30 °C in five 2.5 L flasks containing 1.0 L of MTSB for brevicidine production. The culture supernatant was extracted with Diaion® HP-20 (100 g; Sigma-Aldrich) and eluted with 20% IPA (2.0 L), 40% IPA (2.0 L), 60% IPA (2.0 L), and 80% IPA supplemented with 0.1% trifluoroacetic acid (1.0 L). All partitions were evaporated to dryness using a vacuum concentrator and dissolved in methanol for HPLC purification. The 60% IPA partition was separated using a semi-preparative RP-HPLC column with a gradient of 20–50% MeCN in water to yield compounds **1** (25.0 mg, 5.0 mg L^−1^) and **4**–**6**. *B. laterosporus* ATCC 9141 was cultivated for 2 days at 27 °C in ten flasks with a volume of 2.5 L containing 1.0 L of MTSB for laterocidine (**2**) production. The culture supernatant was extracted with Diaion® HP-20 (100 g) and eluted with 20% IPA (2.0 L), 40% IPA (2.0 L), 60% IPA (2.0 L), and 80% IPA supplemented with 0.1% trifluoroacetic acid (1.0 L). The last two partitions were evaporated to dryness and loaded into a C-18 column, then eluted with 120 mL of 30, 50, 60, 70, 80, 90, and 100% methanol, and 99% methanol supplemented with 1% trifluoroacetic acid. The 50% methanol partition was evaporated to dryness and separated using a semi-preparative RP-HPLC column with a gradient of 20–50% MeCN in water to yield compounds **2** (4.7 mg, 0.47 mg L^−1^) and **8**–**10**.

### Structure elucidation

^1^H, ^1^H-^1^H-COSY, ^1^H-^13^C-HSQC, and ^1^H-^13^C-HMBC NMR spectra for compounds **1**, **2**, and **4** were recorded on a Bruker AV500 spectrometer (500 MHz) using MeOH-*d*_*4*_ (^1^H-NMR MeOH-*d*_4_: *δ*_H_ = 3.31 ppm; MeOH-*d*_4_: *δ*_C_ = 49.00 ppm). Amino acid configurations of **1–2** were determined using the advanced Marfey’s method^36^. To further confirm the amino acid configurations of compounds **1**–**2**, the D-Orn/Lys-specific hydrolysis of **1–2** was performed at 37 °C for 2 h in 25% phosphate-buffered saline (PBS, pH 7.4, 80 μL) with 100 nM D-Orn/Lys-specific peptidase BogQ^28^ and 100 μM substrates (**1**–**2**). Three volumes of cold methanol (240 μL) were added to the samples, which were then incubated at −80 °C for 1 h to precipitate protein. The samples were centrifuged at 21,500 × *g* for 10 min, and the supernatant (2 μL) was injected into the same UPLC-MS system as the one described above. The BogQ hydrolyzed fragments of **1** and **2** were purified using the semi-preparative RP-HPLC column with 25% MeCN in water supplemented with 0.1% trifluoroacetic acid to yield compounds **4**–**7** and **7–10**. The purified compounds **1**–**2** and **4**–**10** (0.1–0.2 mg) were hydrolyzed in 6 M HCl at 120 °C overnight. Each solution was evaporated to dryness under a stream of dry N_2_ and the residue was dissolved in 100 μL of water and divided into two portions. Each portion was treated with 5 μL of NaHCO_3_ (1 M) and 50 μL of 1-fluoro-2, 4-dinitrophenyl-5-L-leucinamide (L-FDLA) or D-FDLA (1 M) at 40 °C for 2 h. The reaction was quenched with 5 μL of HCl (1 M) and diluted with 200 μL of MeOH. The stereochemistry was determined by comparing the L-/D FDLA derivatized samples using UPLC-MS analysis.

### Antibacterial assays

MIC was determined by broth microdilution according to CLSI guidelines. The test medium for most species was Mueller–Hinton broth (MHB). Bacteria were grown overnight to early stationary phase and adjusted in MHB to 5.0 × 10^5^ c.f.u. mL^−1^ in the wells of 96-well microtiter plates (Falcon® 96-Well Flat-Bottom Microplate, Tissue Culture-Treated), mixed with varying concentrations of test compounds and incubated at 37 °C for 24 h. Cell growth was evaluated by measuring the optical density at 595 nm (Thermo Scientific Multiskan FC multiplate photometer) (Waltham, MA, USA), and the MIC was defined as the lowest compound concentration at which no bacterial growth was observed. MHBs containing 10% fetal bovine serum (Gibco), 21 mM MgCl, or 1.0 mg mL^−1^ LPS (lipopolysaccharides from *E. coli* O55:B5, Sigma-Aldrich) was used to test their inhibitory effects.

### Bacterial growth kinetics and time-kill assay

A single fresh colony of *E. coli* TOP10 cells was inoculated into MHB (5 mL) and grown with shaking at 225 rpm overnight. The bacteria were diluted to 1 × 10^5^ c.f.u. mL^−1^ in fresh MHB, grown overnight to early log-phase (OD_600_ = 0.15) and treated with the antibiotics of interest in the wells of a 96-well plate. Final antibiotic concentrations varied from one to eight times the MIC. The plate was then incubated at 30 °C overnight, with OD_600_ measurements taken every 30 s.

An overnight culture of *E. coli* TOP10 cells was diluted 1:10,000 in MHB and incubated at 37 °C with agitation at 180 rpm for 2 h (early exponential). The bacteria were then challenged with brevicidine (20 μg mL^−1^), laterocidine (20 μg mL^−1^), or polymyxin B (10 μg mL^−1^) at ten times the MIC at room temperature without shaking. An untreated sample of cells was used as a negative control. At intervals, 10 μL aliquots were removed, centrifuged at 10,000 × *g* for 1 min and resuspended in 1.0 mL of sterile PBS. Tenfold serially diluted suspensions were plated on MHB agar plates and incubated at 37 °C overnight. Colonies were counted and the number of c.f.u. per mL was calculated. For analysis of lysis, 300 μL of culture (OD_600_ = 1.0) were treated with antibiotics at ten times the MIC for 24 h. Experiments were performed in triplicate.

### Resistance studies

*E. coli* ATCC 25922 was inoculated into MHB overnight at 37 °C with continuous shaking. Cells were diluted 10,000 times in MHB to about 1 × 10^6^ c.f.u. mL^−1^. Ten µL aliquots were drawn to a 96-well plate containing 90 µL of serially diluted brevicidine, laterocidine, and ciprofloxacin, at final concentrations of 0.5×, 1×, 1.5×, 2×, and 4× MIC. Likely due to the bactericidal effects of brevicidine and laterocidine, bacteria could not grow at higher MICs. Plates with bacteria were incubated at 37 °C without shaking. After 24 h, the MIC was recorded, and 1 µL aliquots from the culture with the second-highest antibiotic concentration that showed visible growth were diluted 1000 times in MHB for the subsequent assay. This process was repeated for 30 days, and the final MIC was confirmed by the same antibacterial assay as the one described above. The experiment was performed in quadruplicate, and on each day, the sample that developed the highest resistance was measured to plot the curve. For single-step resistance, *E. coli* ATCC 25922 cells at 10^8^ c.f.u. were plated onto Mueller–Hinton agar containing 4× MIC of brevicidine. After incubation for 48 h at 37 °C, no resistant colonies were detected.

### Cytotoxicity and hemolytic activity

Human HeLa cells were used in the assay. Ninety μL of 1 × 10^5^ mL^−1^ cells were placed into the wells of 96-microwell plates. After 12 h, 10 μL of a medium containing test compounds at various concentrations were added to the cells which were then incubated at 37 °C for 48 h. Afterward, the supernatant was removed and 20 μL of MTT (3-(4,5-Dimethylthiazol-2-yl)-2,5-Diphenyltetrazolium Bromide) (2.5 mg mL^−1^) in PBS were added to each well. After incubation at 37 °C for 3 h, 80 μL of dimethyl sulfoxide (DMSO) were added to each well and incubated for an additional 15 min. The absorbance was then measured at 570 nm with a Thermo Scientific Multiskan FC multiplate photometer.

Hemolytic activity was determined with red blood cells freshly isolated from healthy rabbits. Brevicidine and laterocidine were added at final concentrations of 128, 64, 32, and 16 μg mL^−1^ in 0.5% DMSO to 2% (v/v) erythrocytes in PBS. The cells were incubated for 1 h at 37 °C and centrifuged for 5 min at 10,000 × *g*. The supernatant was transferred to a 96-well plate and the absorbance was measured at a wavelength of 570 nm with a Thermo Scientific Multiskan FC multiplate photometer. The absorbance relative to the positive control, which was treated with 10% Triton X-100, was defined as the percentage of hemolysis.

### Peptide binding to LPS

Microcalorimetric experiments of peptide binding to LPS were performed on an MCS isothermal titration calorimeter (Microcal, Northampton, MA) at 37 °C. LPS was prepared as a 50 µM aqueous suspension in 20 mM HEPES buffer (pH 7.0) by suspension, sonication, and temperature cycling between 5 and 70 °C. This suspension was stored at 4 °C overnight prior to use. Aliquots of 100 µM peptide solutions (brevicidine and polymyxin B) were prepared in the same buffer and all solutions were degassed prior to use by stirring under vacuum for 5 min at 37 °C. After thermal equilibration, 5 µL aliquots of the peptide solution were added every 3 min into the lipid-containing cell at 37 °C with constant stirring. The change in heat during the titration steps was registered in real time and raw data were processed using the Origin® 7 software provided with the instrument. In control experiments, the corresponding peptide solution (or LPS solution) was injected into the buffer without LPS (or without peptide). Heats of dilution were significantly lower than those during ligand-receptor binding.

### Atomic force microscopy imaging of bacterial cells

One hundred μL of log-phase *E. coli* ATCC 25922 cells (OD_600_ = 0.2) in an LB medium were incubated for 1.5 h at 37 °C with or without the test compounds at ten times the MIC. The samples were then centrifuged at 8000 × *g* for 10 min at 4 °C, and the pellet was washed twice in 100 μL of apyrogenic water. The bacteria resuspended in 50 μL of apyrogenic water were applied to mica disks and dried overnight at 28 °C before imaging. Atomic force micrographs were recorded on a Veeco Mulitmode AFM with NanoScope III controller operating in contact mode. The data were analyzed with NanoScope Analysis software v.1.40 (Veeco, USA). Scans were acquired at 25 °C at the rates of 1.0 Hz and 256 samples per line resolution. Downstream image processing and analysis were performed using NanoScope software. Height images were flattened to compensate for cell curvature, and topographical sections were used to reconstruct the surface texture in two dimensions.

### Mouse thigh infection model

In vivo activities of brevicidine and laterocidine were tested using the mouse thigh infection model^37^. Animal studies were performed by Pharmacology Discovery Services Taiwan Ltd. in general accordance with standard guidelines on animal welfare^38^ in an AAALAC-accredited facility. The protocol was reviewed and approved by the Institutional Animal Care and Use Committee. Groups of five female-specific pathogen-free ICR (CD-1) mice weighing 22 ± 2 g (~5 weeks of age) were used. Animals were immunosuppressed with two intraperitoneal injections of cyclophosphamide, the first at 150 mg kg^−1^ 4 days before infection (day −4) and the second at 100 mg kg^−1^ 1 day before infection (day −1). On day 0, animals were inoculated intramuscularly (0.1 mL per thigh) in the right thigh with an *E. coli* ATCC 25922 suspension. Vehicle (10% DMSO, 1% Tween 80, 0.9% NaCl) and antibiotics were then subcutaneously injected 2 and 8 h later. Due to the limited availability of compounds **1**–**2**, only one concentration of **1** (30 mg kg^−1^) or **2** (15 mg kg^−1^) was used in the present study. Precisely 26 h after inoculation, animals were euthanized by CO_2_ asphyxiation before their right thigh muscles were harvested. The removed muscles were homogenized in 5 mL of PBS, pH 7.4, with a Polytron homogenizer. Homogenates in the amount of 0.1 mL were used for serial tenfold dilutions and plated onto a nutrient agar medium for colony count (in c.f.u. g^−1^) determination. No randomization or blinding was necessary for the animal infection models, and no samples were excluded.

### Gene deletion using the CRISPR-Cas9 system

Vector pJOE8999-*latC* was constructed for deleting *gene latC* in the chromosome of *B. laterosporus* ATCC 9141 using the CRISPR-Cas9 system^26–28^. In the first step, the plasmid pJOE8999 was cut with *Bsa*I and the *lacZ* α fragment was replaced by the two complementary oligonucleotides *LatC*-sgRNA-F/R. In the second step, two fragments flanking *latC* ~400 bp in length were amplified by polymerase chain reaction (PCR) using primers *LatC*-Up-F/R and *LatC*-41-Do-F/R from *B. laterosporus* ATCC 9141 chromosomal DNA as the template, and then cut with SfiI and inserted between the two SfiI sites to give pJOE8999-*latC*. Plasmid pJOE8999-*latC* was transformed into *B. laterosporus* ATCC 9141 according to a modified electroporation method^28^. Briefly, these competent cells mixed with 200 ng of the pJOE8999-*latC* vector DNA were pulsed using a Gene-Pulser electroporation system and then plated on selective LB agar plates containing 25 mg L^−1^ kanamycin and 0.2% mannose for induction of *cas9*. The positive transformants were then inoculated at 22 °C for 2 days on selective LB agar plates. The positive colonies with knockout vector were streaked onto LB plates without antibiotics and incubated at 45 °C for plasmid curing. The colonies cured of plasmids were confirmed by streaking them onto LB plates containing kanamycin (25 mg L^−1^), where they failed to grow at 37 °C. The mutant colonies without plasmid were confirmed by colony PCR using the outer primers from the homology templates.

### Code availability

Scripts for BGC analysis and networking analysis are provided in Supplementary Data 1.

### Data availability

All data sets generated or analyzed in the current study have either been included in this published article (and its Supplementary Information files) or are available from the corresponding author on request.

## Electronic supplementary material

Supplementary Information

Description of Additional Supplementary Files

Supplementary Data 1
