# Supplementary material for: Discovery of cationic nonribosomal peptides as Gram-negative antibiotics through global genome mining
Source: Nat Commun. 2018 Aug 16;9:3273. doi: 10.1038/s41467-018-05781-6 (PMC6095874; doi:10.1038/s41467-018-05781-6)
Supplement: Supplementary file 1 — Supplementary Information [file 41467_2018_5781_MOESM1_ESM.pdf]

Discovery of cationic nonribosomal peptides as Gram-negative antibiotics through global genome mining

Yong-Xin Li,[1, 2,3] Zheng Zhong,[1, 3] Wei-Peng Zhang,[1] and Pei-Yuan Qian[1]*

Affiliations

[1] Division of Life Science and Department of Ocean Science, Hong Kong University of Science and Technology, Clear Water Bay, Hong Kong, China

[2] Institute for Advanced Study, Hong Kong University of Science and Technology, Clear Water Bay, Hong Kong, China

[3]These authors contributed equally to this work.

* e-mail: boqianpy@ust.hk

Table of Contents

Supplementary Tables 1-2

Supplementary Figures 1-15

Supplementary Notes (MS/NMR data)

Supplementary Table 1. Antimicrobial activities of CNRPs and their derivatives.

	Bacterial strains (MIC/μg.mL^{-1})								
Comp.	*Ec*	*Pa*	*Ab*	*Kp*	*En*	*Bs*	*MRSA*	*Ca*	*Sc*
**1**	2	1	16	2	**2**	32	ND	ND	ND
**2**	2	2	4	4	**2**	32	ND	ND	ND
**4**	ND	ND	ND	ND	ND	ND	ND	ND	ND
**5**	ND	ND	ND	ND	ND	ND	ND	ND	ND
**6**	ND	ND	-	-	-	ND	ND	-	-
**7**	ND	ND	-	-	-	ND	ND	-	-
**8**	ND	ND	-	-	-	ND	ND	-	-
**9**	ND	ND	-	-	-	ND	ND	-	-
**10**	ND	ND	-	-	-	ND	ND	-	-
**11**	16	8	-	-	-	ND	ND	-	-
Polymyxin	1	1	4	16	**8**	4	32	ND	ND

Ec = Escherichia coli ATCC 25922, *Pa = Pseudomonas aeruginosa* PAO1, *Ab = Acinetobacter baumannii*, *Kp = Klebsiella pneumoniae NRRL-B-408, En = Enterobacter cloacae* NRRL-B-413, *Bs* = *B. subtilis* 168, *MRSA* = Methicillin-resistant *Staphylococcus aureus* ATCC 43300*, Ca = Candida albicans, Sc = Saccharomyces cerevisiae VL-48*, ND>64 μg mL^{-1}.

Supplementary Table 2. Predicted amino acids residues and their assigned short forms.

**Predicted amino acids**	**Assigned amino acids**
ala, abu, alaninol, b-ala, dhb, ala-d, apa, cha	A
cys	C
asp, measp, omasp	D
glu, 3mglu, aaa, ahp, aad, aeo	E
phe, phenylacetate, qa, sal, phg	F
gly, sar	G
his	H
ile	I
lys, 2-3-diaminoproprionate, blys, dab, orn, pip	K
leu	L
met	M
asn, hasn	N
pro, mpro, apc	P
gln, uda	Q
arg, capreomycidine, guanidinoacetic_acid, cit, end, gua	R
ser, hse	S
thr, bmt, thr-4-cl, tcl	T
val, hyv	V
trp	W
tyr, 3-hpa, 4-mha, bht, dhpg, homotyr, hpg, hpg2cl, kyn, qna, dpg, hty	Y

Both proteogenic and non-proteogenic amino acids predicted by antiSMASH were assigned to their short forms according to this table. Non-proteinogenic amino acids were assigned to proteinogenic counterpart based on their structure similarity or biosynthesis origin.

Supplementary Figure 1

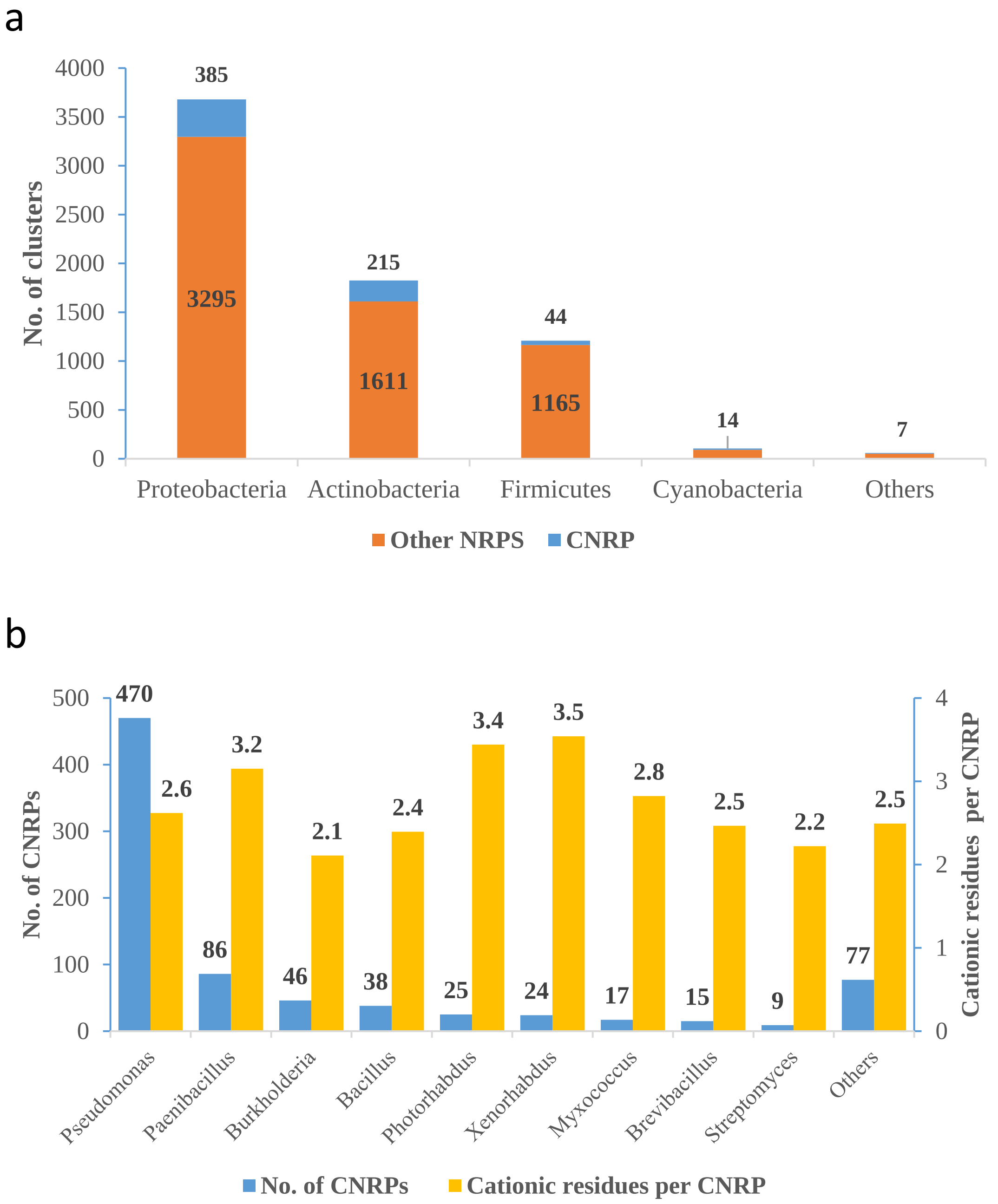

Supplementary Figure 1. The phylogenetic distribution of CNRP BGCs in bacteria revealed by global genome mining. a) Global BGCs analysis of bacteria complete genomes (5,585) revealed that CNRPs (665) were abundantly distributed in Proteobacteria, Actinobacteria and Firmicutes. **b**) The wide distribution of CNRP BGCs at the genus level. To further investigate the biosynthesis capacity of CNRPs, 1810 draft genomes from top 20 genera abundant in CNRPs were also selected for BGCs analysis. In total, CNRP BGCs obtained from 7,395 bacterial genomes were filtered and subjected to sequence similarity study as showed in Fig. 1.

Supplementary Figure 2

a

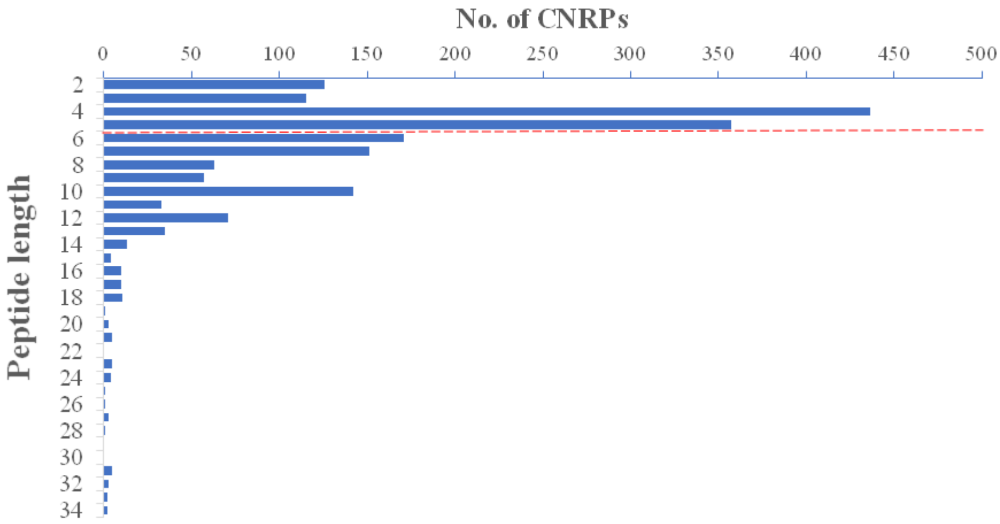

b

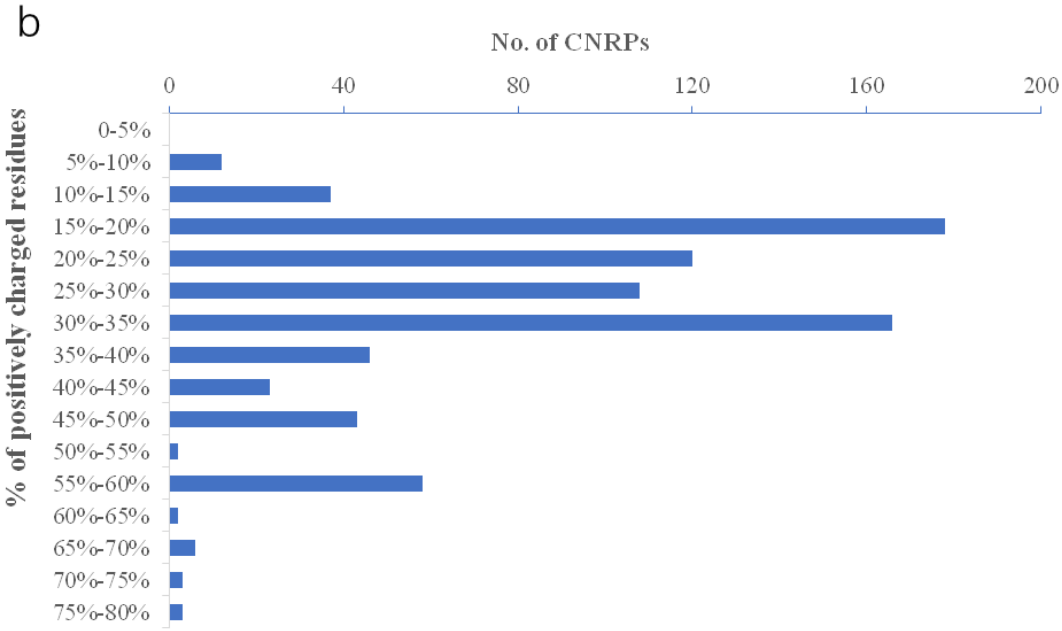

Supplementary Figure 2. The diversity of CNRPs revealed by global genome mining of 7,395 bacterial genomes. a) The diversity of CNRPs in terms of peptide length. The length of CNRPs (n = 1,818) varied from 2 to 34, with an average of 6.5 and a median of 5, which indicated their high diversity. Below the red dotted line (length ≥ 6) are CNRPs (n = 807) that exist in the similarity network in Fig.1. These CNRPs have an average length of 9.7 and a median length of 9. **b)** The diversity of CNRPs in positively-charged residues percentage. The percentage of positively-charged residues arranged from 6.3% to 77.8% with an average of 30.6% (median 28.6%).

Supplementary Figure 3

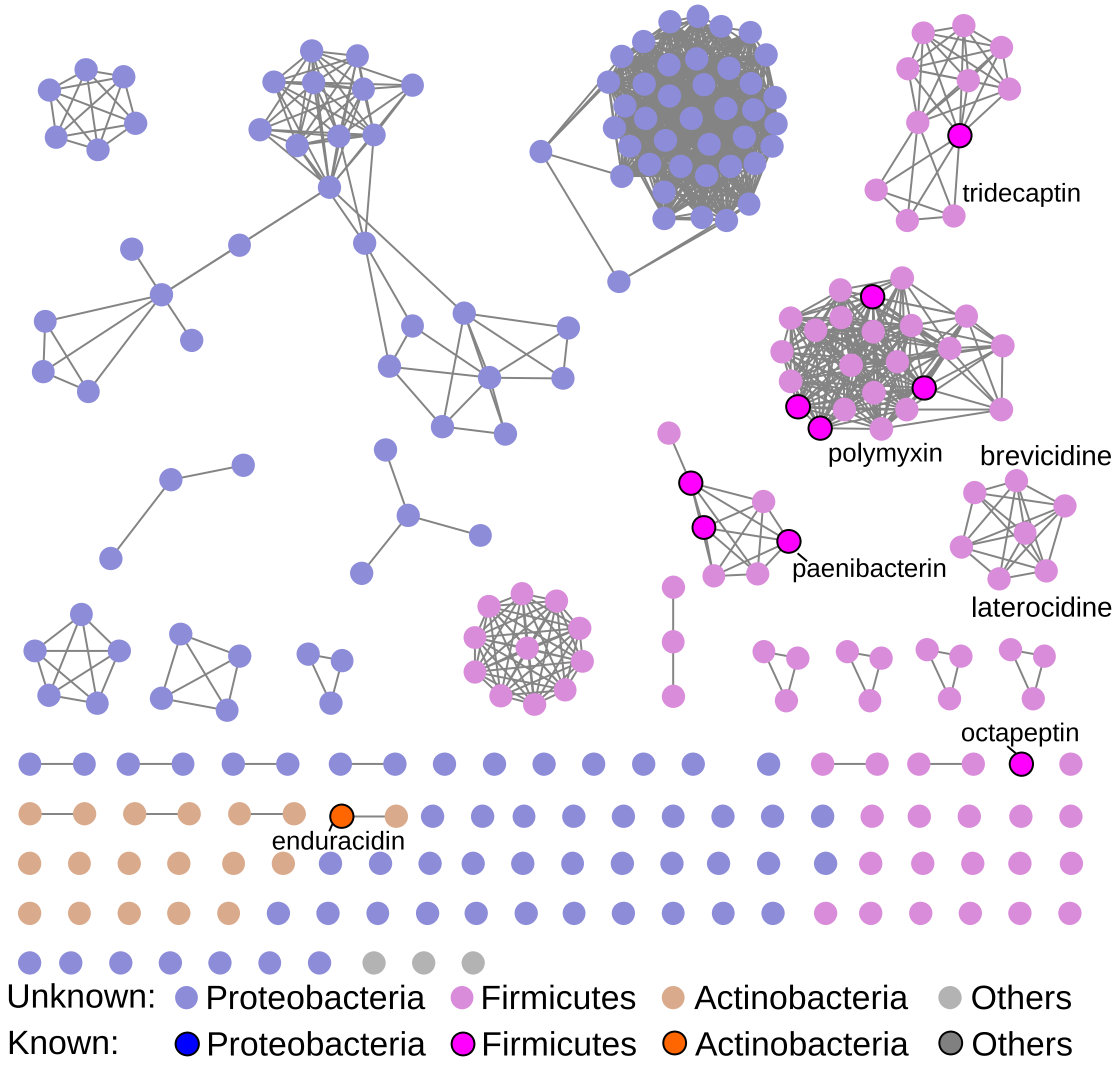

Supplementary Figure 3. A sequence similarity network of N-acylated CNRPs (cationic lipopeptides) showing their diversity, distribution, and discovery status. Clearly, the majority of N-acylated CNRPs remain unexplored.

Supplementary Figure 4

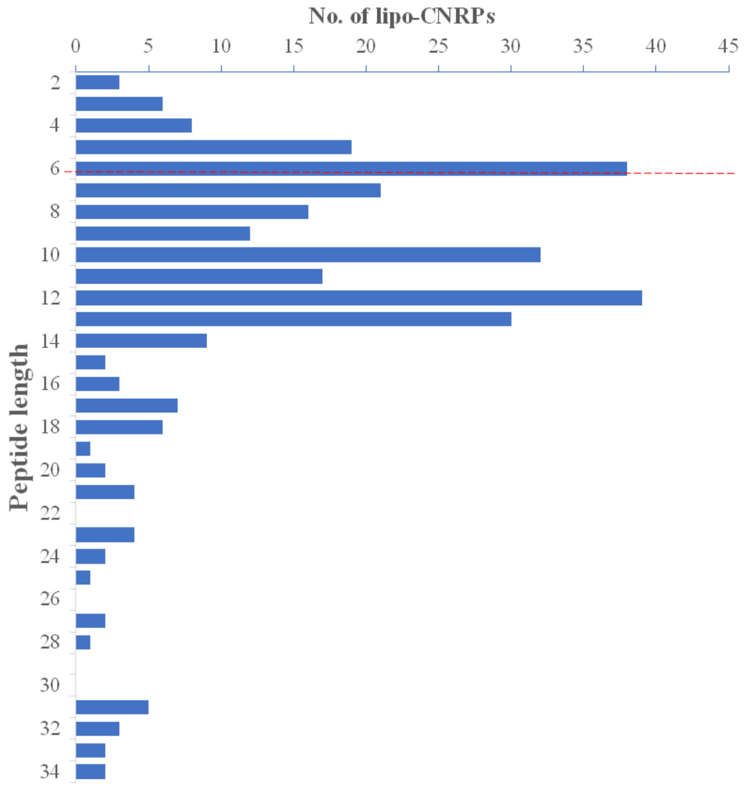

Supplementary Figure 4. The diversity of putative N-acylated CNRPs (cationic lipopeptides) in terms of peptide length. CNRPs with a condensation starter domain (Cs) in their BGCs were considered as N-acylated CNRPs. The length of N-acylated CNRPs ($n = 297$) varied from 2 to 34, with an average of 11.2 and a median of 10. Below the red dotted line (length ≥ 6) are N-acylated CNRPs ($n = 261$) that exist in the sequence similarity network (See Supplementary Figure 3). These N-acylated CNRPs have an average length of 12.19 and a median length of 11.

Supplementary Figure 5.

a

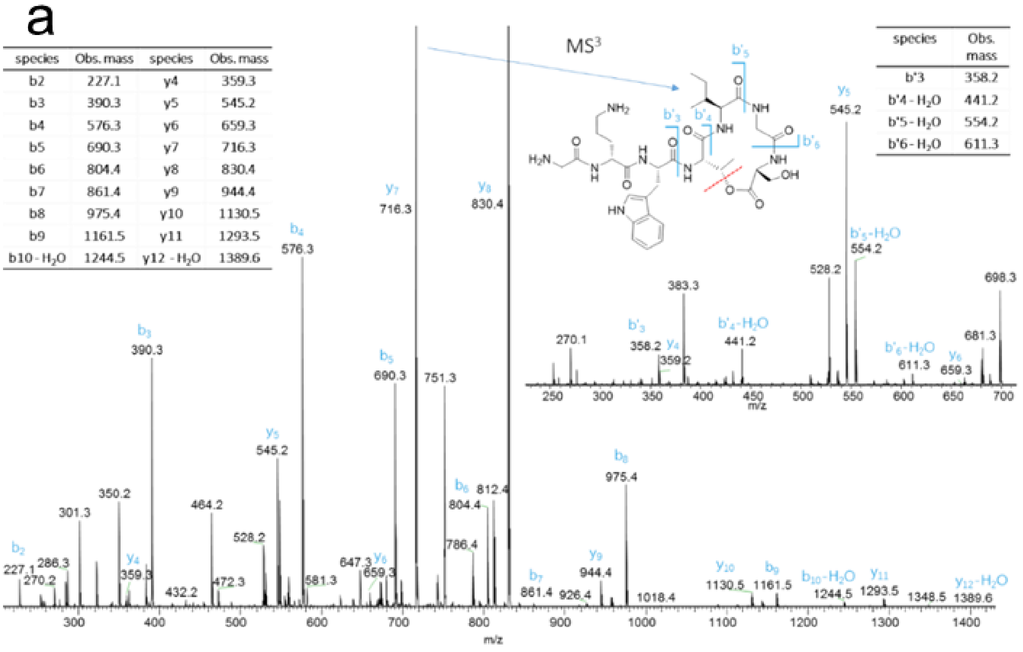

b

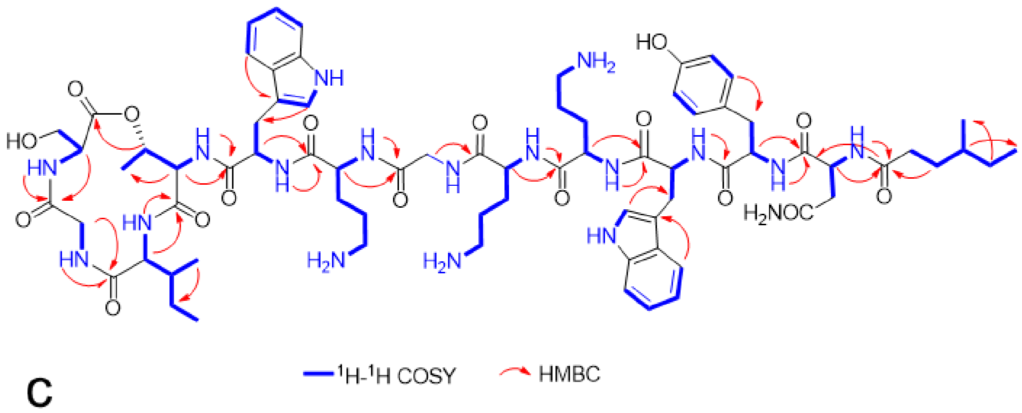

c

Amino Acid Residue	1		6		7		4		Configuration
	D-FDLA (D)	L-FDLA (L)	D	L	D	L	D	L	
Asn_1	10.4	10.8	10.4	10.8	-	-			D
Tyr_2 (di)	15.2	16.1	15.2	16.1	-	-			D
Trp_3	12.7, 13.2	12.7, 13.2	12.7	13.2					D
Trp_8							13.2	12.7	L
Orn_4	8.6, 9.2	8.6, 9.2	9.2	8.6					D
Orn_5					8.6, 9.2	8.6, 9.2			L
Orn_7									D
Thr_9	11.2	10.3					11.2	10.3	L
Ile_{10}	14.5	12.6					14.5	12.6	L
Ser_{12}	10.7	10.4					10.7	10.4	L

Supplementary Figure 5. MS, NMR and Marfey-type analyses of brevicidine (1).

a) MS_n analysis of **1**. **b**) The key ^1H-^1H-COSY and ^1H-^{13}C-HMBC correlation of

compounds **1**. **c**) Marfey-type analysis of brevicidine (Retention times (in min) of **1** constituent amino acids derivatized with D/L-FDLA). The ^{1}H, ^{1}H-^{1}H-COSY, ^{1}H-^{13}C-HSQC, and ^{1}H-^{13}C-HMBC NMR data of **1** were recorded on a Bruker AV500 spectrometer (500 MHz) using DMSO-d_6 (^{1}H-NMR MeOH-d_4: δ=3.31 ppm; DMSO-d_6: δ=2.50 ppm; ^{13}C-NMR: MeOH-d_4: δ=49.00 ppm; DMSO-d_6: δ=39.8 ppm) (Supplementary Table 3).

Amino acid configurations of **1** were determined using the advanced Marfey's method. The compound **1** and its hydrolytic products **4, 6** and **7** (0.1-0.2 mg) were hydrolyzed in 6 M HCl at 110 °C overnight. Each solution was evaporated to dryness and the residue was dissolved in 100 µL water and divided into two portions. Each portion was treated with 20 µL NaHCO3 (1M) and 50 µL 1-fluoro-2, 4-dinitrophenyl-5-L-leucinamide (L-FDLA) or D-FDLA (1M) at 40 °C for 2 h. The reaction was quenched with 5 µL HCl (1M) and diluted with 200 µL MeOH. The stereochemistry was determined by comparison of the L-/D FDLA derivatized samples using UPLC-MS analysis. On the basis of the combination of the data generated by advanced Marfey's analysis of four compounds, a lipodepsipeptide 4-Methyl-Hexanoyl-D-Asn-D-Tyr-D-Trp-D-Orn-Orn-Gly-D-Orn-Trp-Thr-Ile-Gly-Ser (cyclized via lactone formation between Thr and the C-terminus) was established for the natural product brevicidine. The structure of **1** is consistent with prediction based on biosynthetic pathway analysis (Supplementary Fig. 10).

Supplementary Figure 6

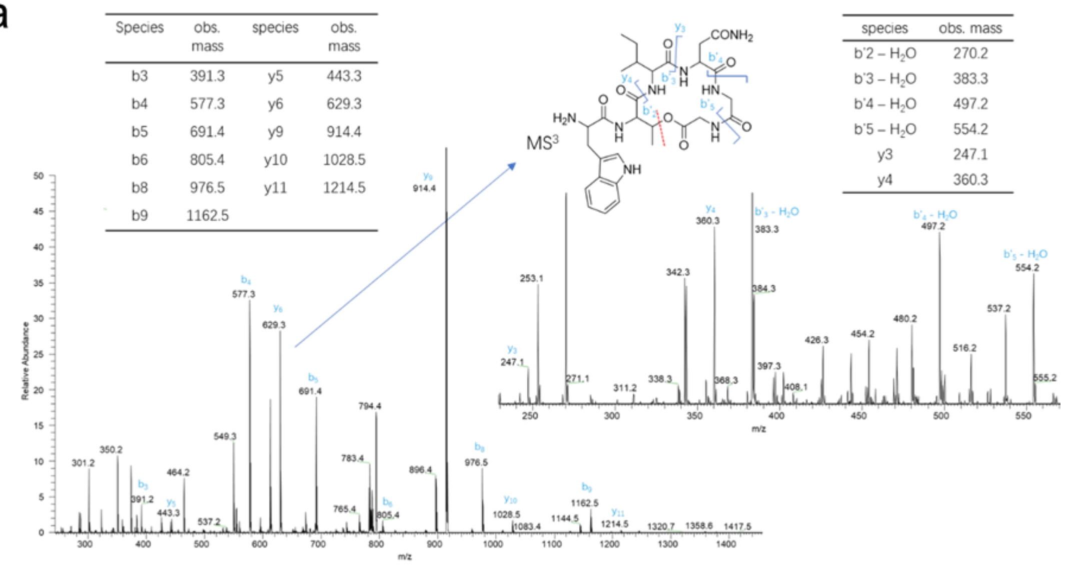

b

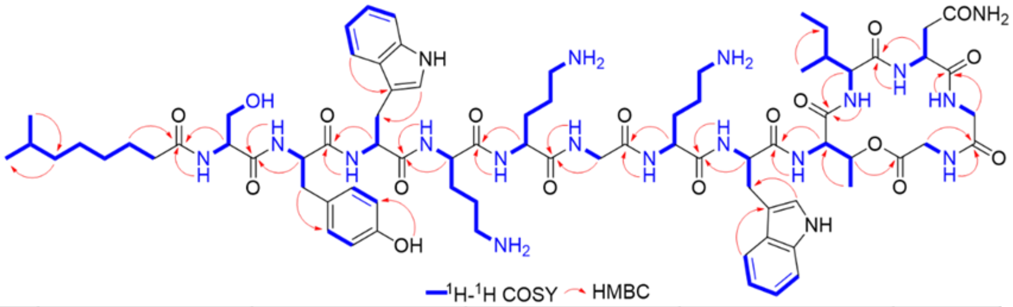

c

Amino Acid Residue	2		10		7		8		Configuration
	D-FDLA (D)	L-FDLA (L)	D	L	D	L	D	L	
Ser_1	10.4, 10.6	10.6	10.4	10.6	-	-			D
Tyr_2 (di)	15.1, 15.6, 15.9	15.9	15.1, 15.6, 15.9	15.9	-	-			D
Trp_3	12.7, 13.2	12.7, 13.2							D
Trp_8									L
Orn_4	8.6, 9.2	8.6, 9.2	8.6, 9.2	8.6					D
Orn_5					8.6, 9.2	8.6, 9.2			L
Orn_7									D
Thr_9	11.4, 10.3	10.3					11.4, 10.3	10.3	L
Ile_{10}	14.4, 12.5	12.5					14.4, 12.5	12.5	L
Asn_{11}	10.8, 10.5	10.5					10.8, 10.5	10.5	L

Supplementary Figure 6. MS, NMR and Marfey-type analyses of of lacterocidine (2). a) MS_n analysis of laterocidine. **b**) The key ^1H-^1H-COSY and ^1H-^{13}C-HMBC correlation of compounds **2**. **c**) Marfey-type analysis of brevicidine (Retention times (in min) of **2** constituent amino acids derivatized with D/L-FDLA). The ^1H, ^1H-^1H-COSY, ^1H-^{13}C-HSQC, and ^1H-^{13}C-HMBC NMR data of **2** were recorded on a Bruker AV500 spectrometer (500 MHz) using DMSO-*d6* (^1H-NMR: DMSO-*d6*: δ=2.50 ppm; ^{13}C-NMR: DMSO-*d6*: δ=39.8 ppm). Compound **2** was obtained as a white amorphous solid. Based on HRESIMS data (*m/z*: 802.9345 $[M+2H]^{2+}$, calc 802.9329), we

established its molecular formula as $C_{78}H_{113}N_{19}O_{18}$. The characteristic signals of the amide NH and α-amino proton in the ^{1}H NMR spectrum and carbonyl groups in the ^{13}C NMR spectrum (Supplementary Table 4) indicated the peptidic nature of the compound. The gross structure of laterocidine was further established by analyses of the ^{1}H, ^{13}C, ^{1}H-^{1}H COSY, HMQC, and HMBC NMR spectral data (Supplementary Fig. 6b and Supplementary Notes), revealing the presence in its structure of Orn (3), Trp (2), Gly (3), Ile (1), Ser (1), Thr (1), Asn(1), Tyr (1) and a lipidic residue that was identified as 7-methyloctanoic acid. The gross structure established by NMR analysis was also corroborated by MS/MS analysis (Supplementary Fig. 6a).

Amino acid configurations of **2** were determined through Marfey's analysis of compound **2** and its hydrolytic products **7**, **8** and **10** as described above. On the basis of the combination of the data generated by advanced Marfey's analysis and biosynthetic pathway analysis (Supplementary Fig.9), a lipodepsipeptide 7-Methyl-Octanoyl-D-Ser-D-Tyr-D-Trp-D-Orn-Orn-Gly-D-Orn-Trp-Thr-Ile-Asn-Gly-Gly (cyclized via lactone formation between Thr and the C-terminus) was established for the natural product laterocidine.

Supplementary Figure 7.

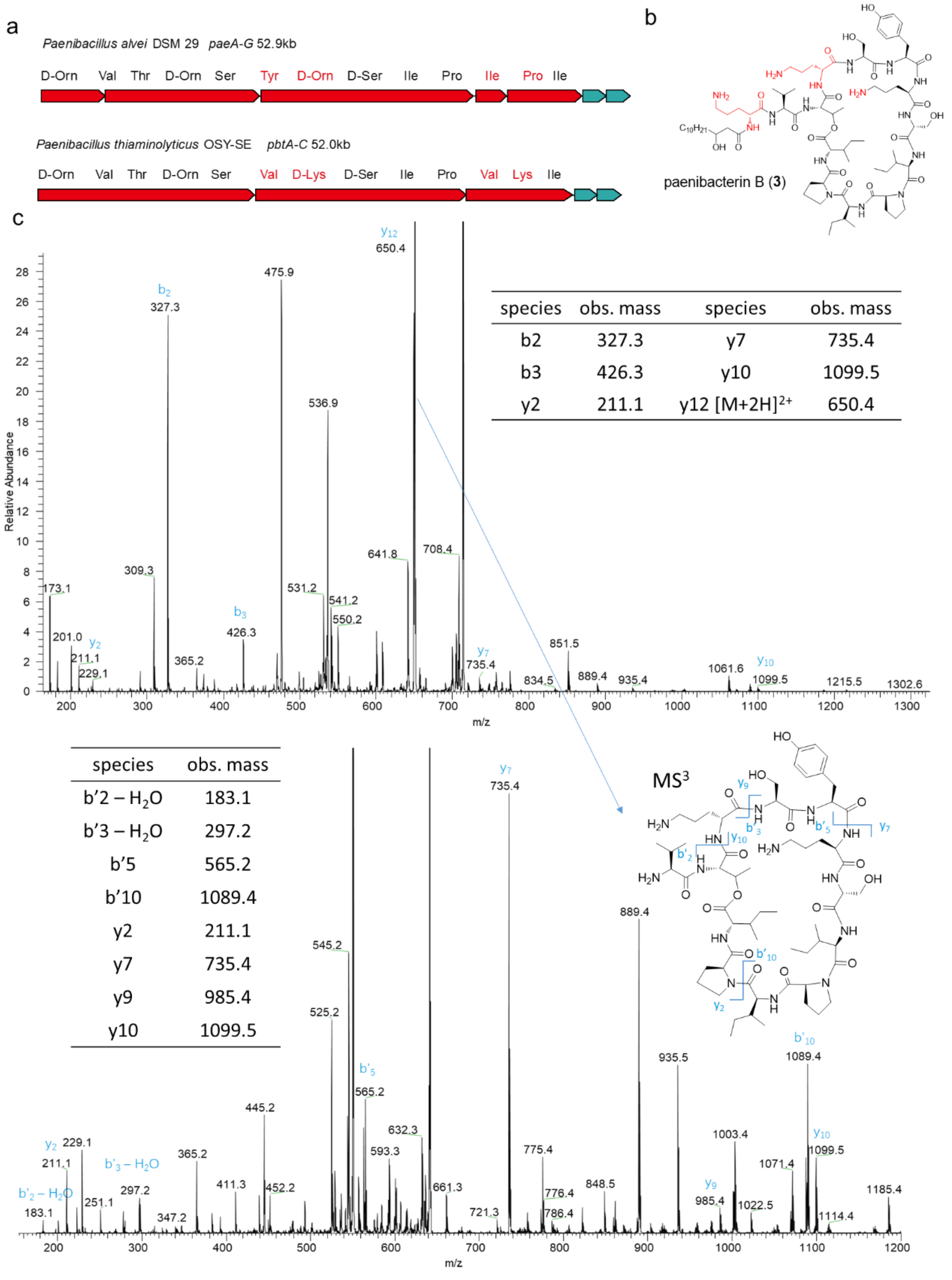

Supplementary Figure 7. MS analyses of Paenibacterin B (3). a) Comparison of BGCs of paenibacterin B (*paeA-G,* from NZ_AMBZ01000008, 57,959-110,917nt)) and paenibacterin A (*pbtA-C*). **b)** Structure of paenibacterin B, identified by MS/MS analysis. **c)** MS_n analysis of paenibacterin.

Supplementary Figure 8.

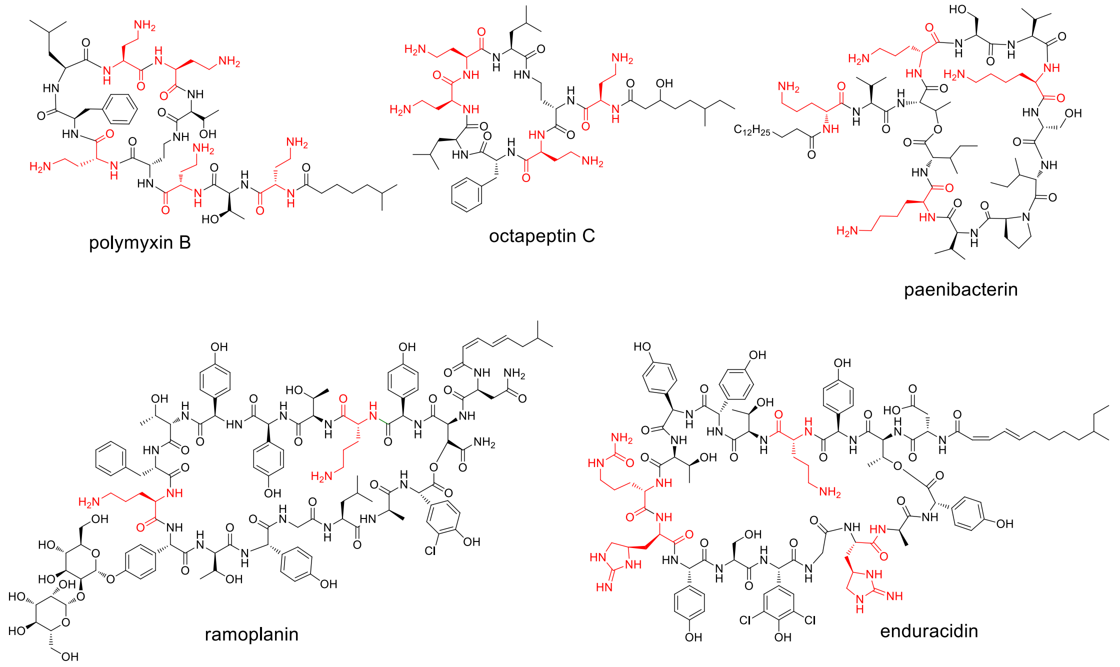

Supplementary Figure 8. Representative members of cyclic cationic lipopeptides. Positively-charged amino acids were highlighted in red.

Supplementary Figure 9

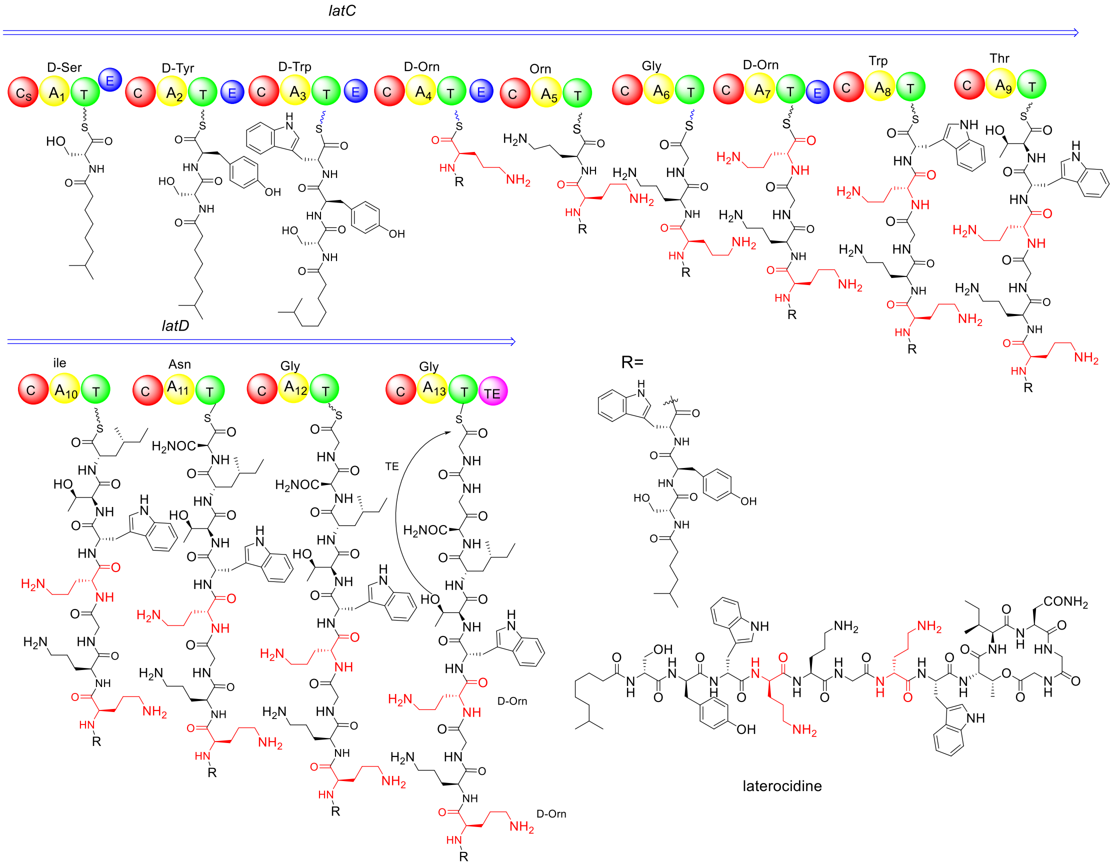

Supplementary Figure 9. The predicted biosynthetic pathway of laterocidine (Domain abbreviation, C, condensation; A, adenylation; T, thiolation (carrier); E, epimerization; and TE, thioesterase; gene cluster location: NZ_CP007806.1, 2,835,471-2,890,522) Biosynthesis of laterocidine starts with the typical starting module for NRPs with a condensation starter domain that acylates the N-terminal with a fatty acid. In accordance with the co-linearity rule, 13 amino acids are introduced into the linear peptide, and the ring closure between the last Gly_{13} and Thr_{9} is catalyzed by the thioesterase domain during product offloading, resulting in laterocidine.

Supplementary Figure 10

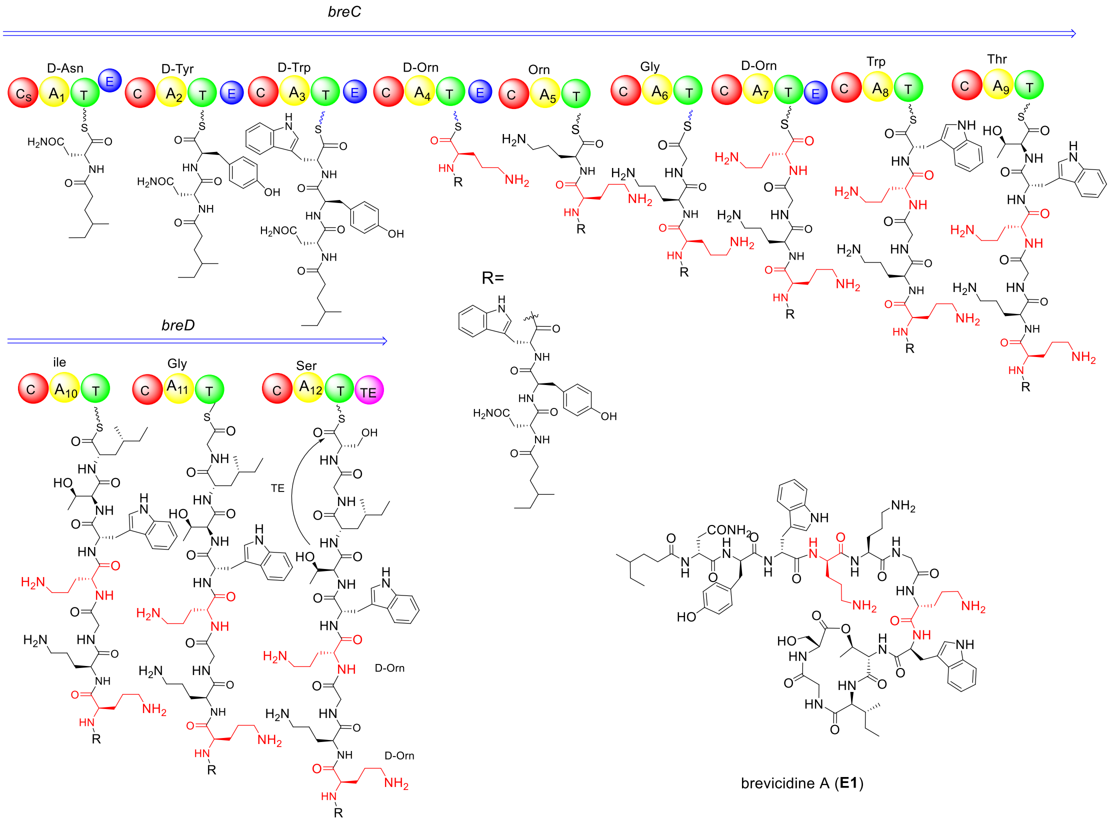

Supplementary Figure 10. The predicted biosynthetic pathway of brevicidine (Domain abbreviation, C, condensation; A, adenylation; T, thiolation (carrier); E, epimerization; and TE, thioesterase; gene cluster location: KB894285, 144,281-196,331nt) Biosynthesis of brevicidine starts with the typical starting module for NRPs with a condensation starter domain that acylates the N-terminal with a fatty acid. In accordance with the co-linearity rule, 12 amino acids are introduced into the linear peptide, and the ring closure between the last Ser_{12} and Thr_9 is catalyzed by the thioesterase domain during product offloading, resulting in brevicidine.

Supplementary Figure 11.

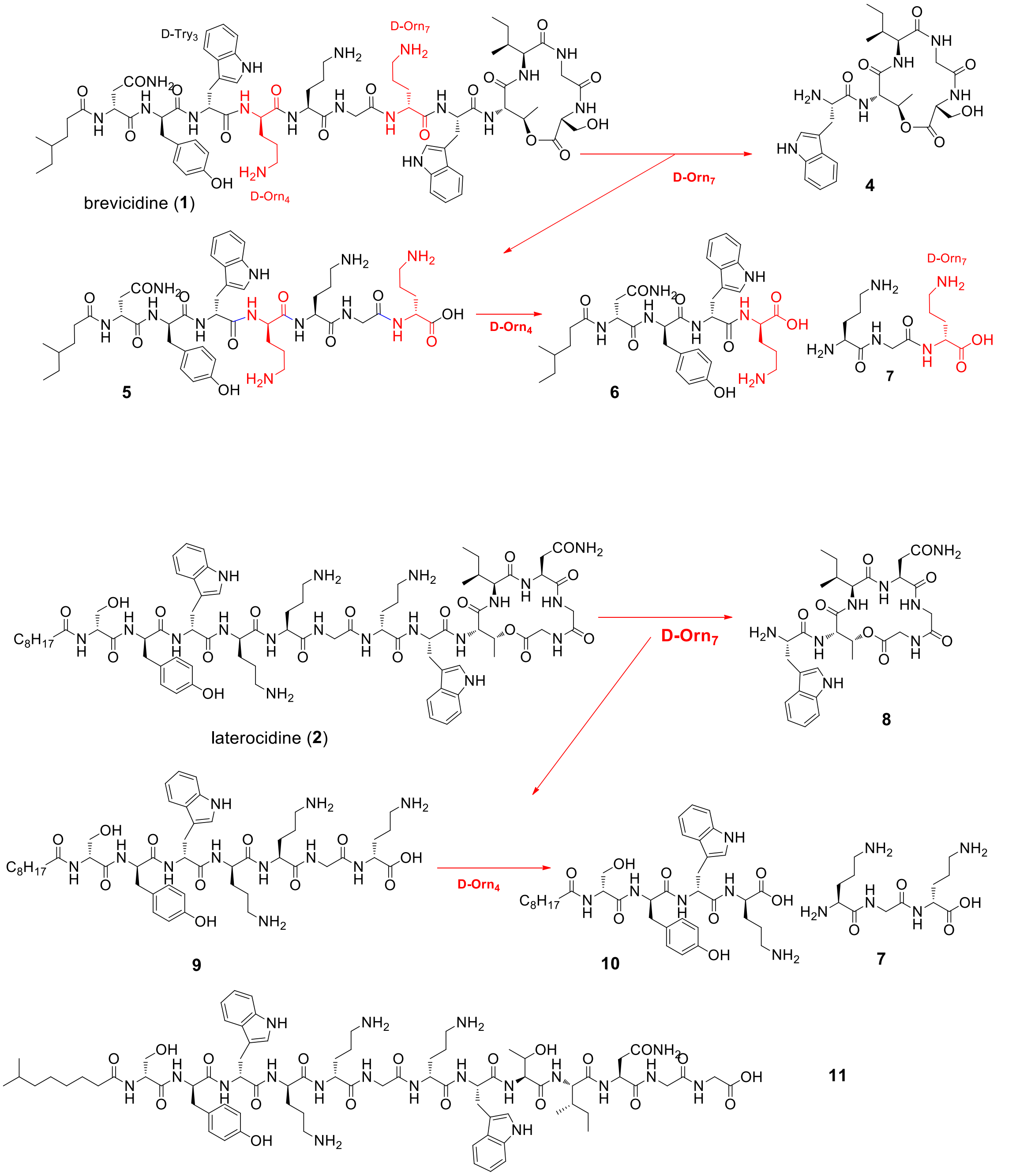

Supplementary Figure 11. Chemical structure of CNRP derivatives.

Supplementary Figure 12

a

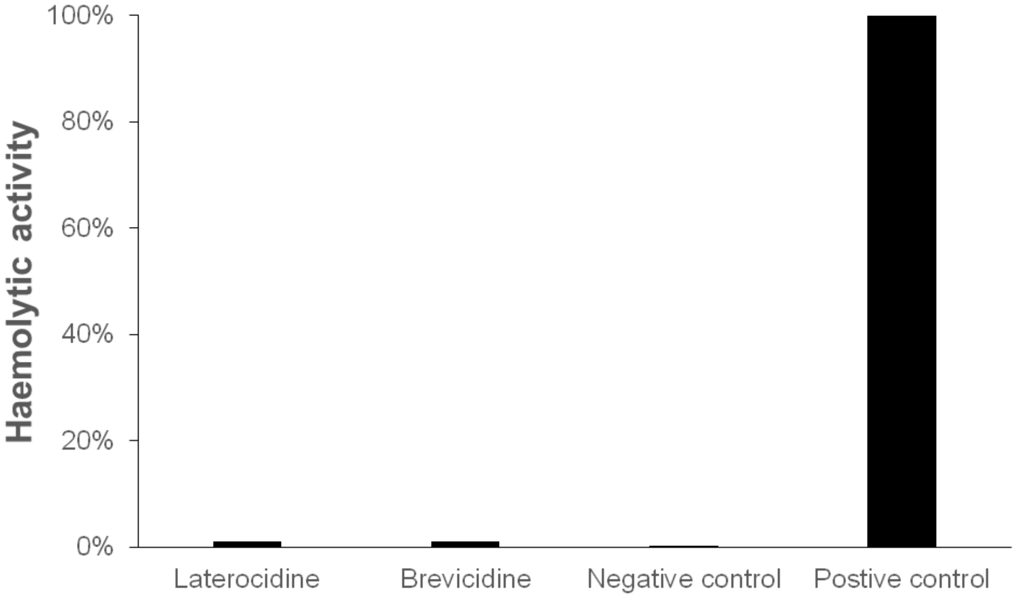

b

Compound	Human HeLa cells IC_{50} (µg mL^{-1})
brevicidine	>128
laterocidine	>128
bogorol	<8

Supplementary Figure 12. Brevicidine and laterocidine activity against eukaryotic cells. a) Rabbit erythrocytes were incubated with brevicidine or laterocidine at concentrations up to 128 µg mL^{-1}. Their lysis was monitored by the release of hemoglobin. Cells without a tested compound were used as negative control. Incubation of cells in 10% Triton X-100 was used as positive control for complete lysis. The data are representative of two independent experiments. **b**) Cytotoxicity of brevicidine and laterocidine. The data are representative of three independent experiments.

Supplementary Figure 13.

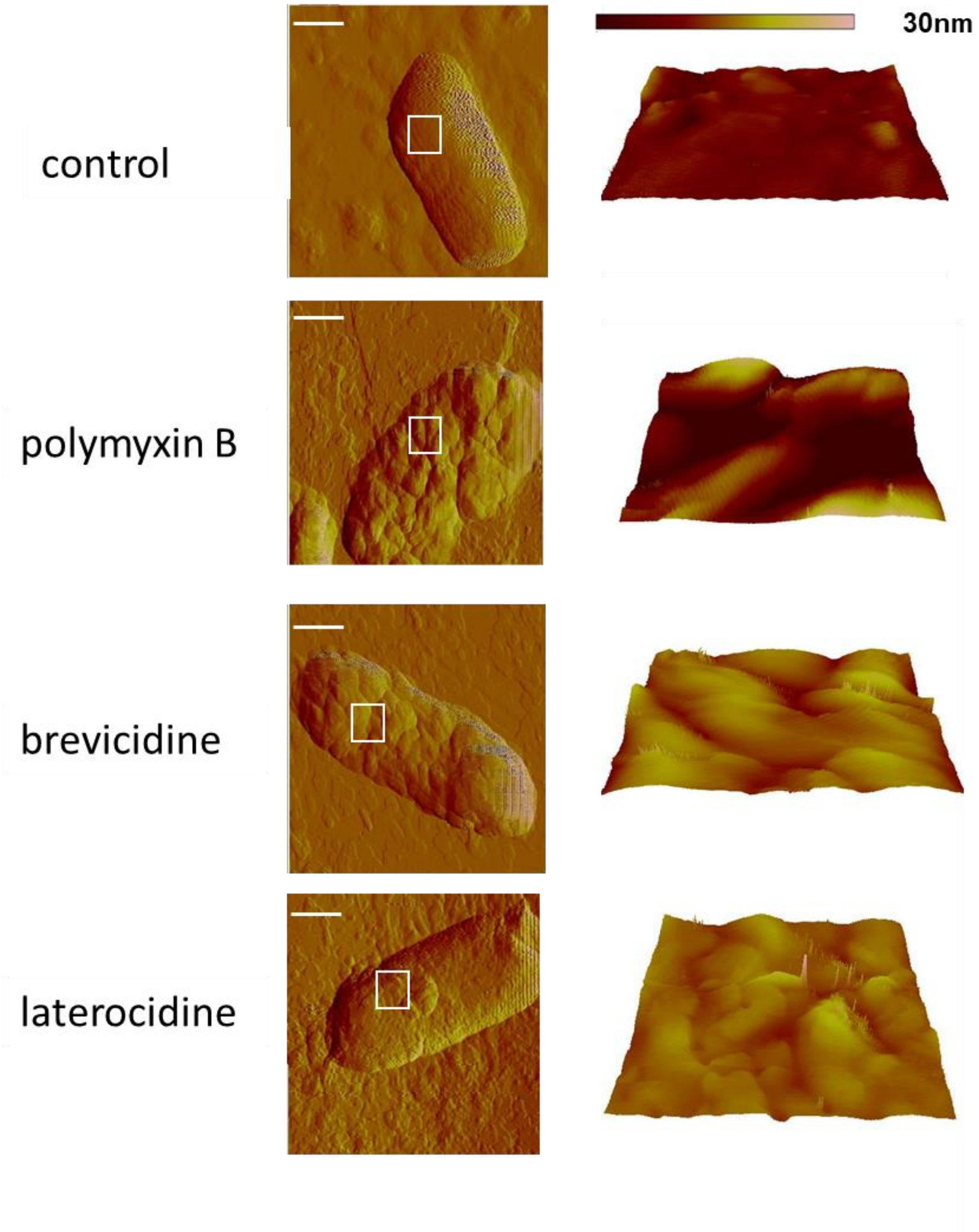

Supplementary Figure 13. Atomic force microscopy of brevicidine/laterocidine-treated *E. coli* ATCC 25922 revealed a dramatic effect on the outer membrane structure. The white square highlights the region scanned to obtain high-resolution topographical images. The data are representative of two independent experiments. Scale bars are 1 μm.

Supplementary Figure 14.

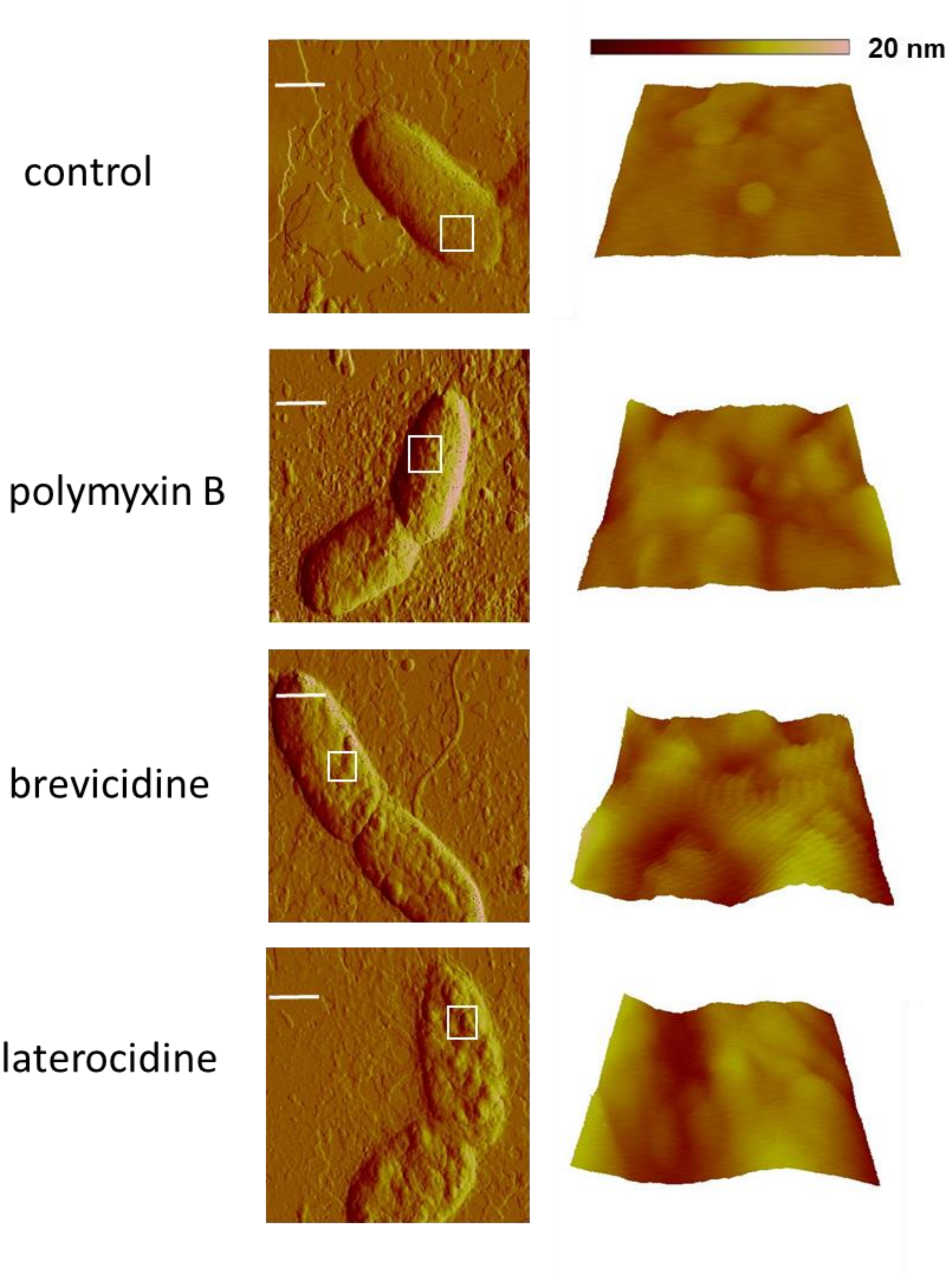

Supplementary Figure 14. Atomic force microscopy of brevicidine/laterocidine-treated *P. aeruginosa* PAO1 revealed a dramatic effect on the outer membrane structure. The white square highlights the region scanned to obtain high-resolution topographical images. The data are representative of two independent experiments. Scale bars are 1 μm.

Supplementary Figure 15.

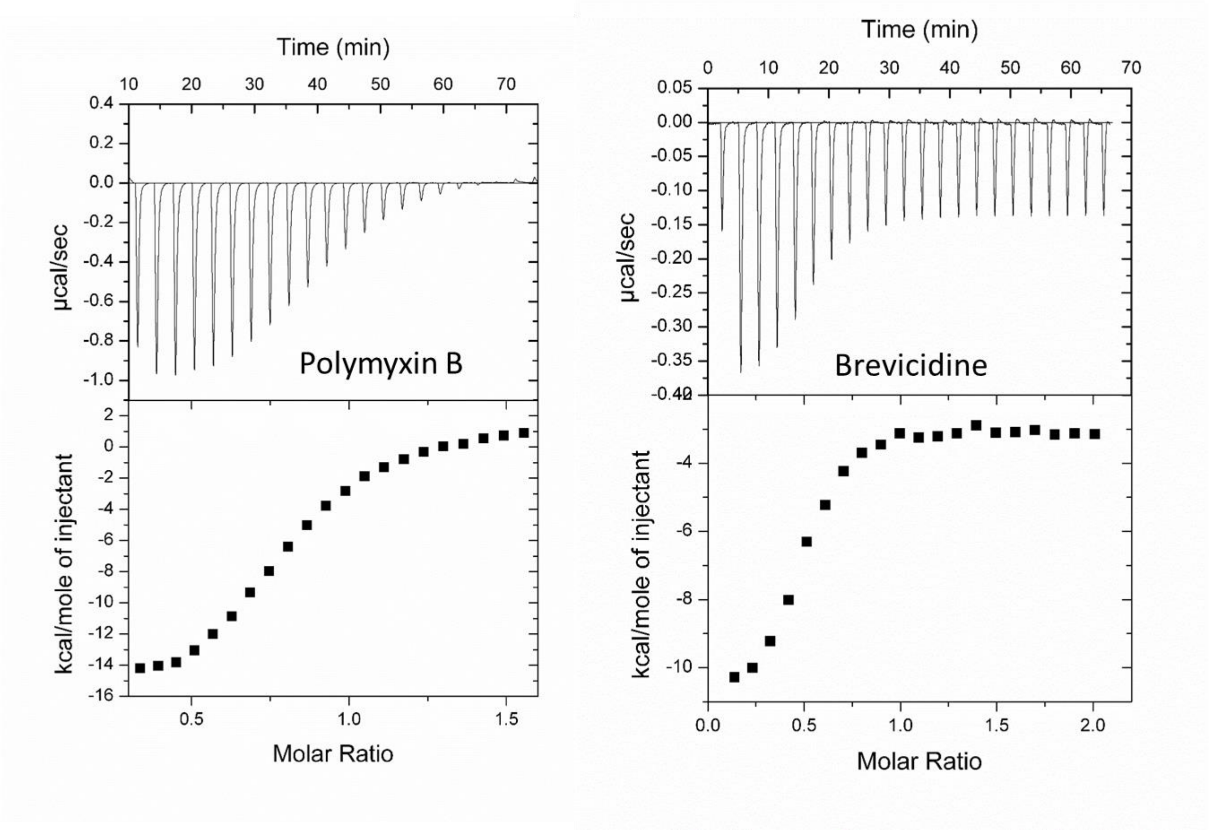

Supplementary Figure 15. Binding of brevicidine and polymyxin B to LPS from *E. coli* revealed by isothermal titration calorimetry (ITC) experiment. **a**) Polymyxin B + LPS. **b**) Brevicidine + LPS. All results show strong binding to LPS.

Supplementary Notes (MS/NMR data)

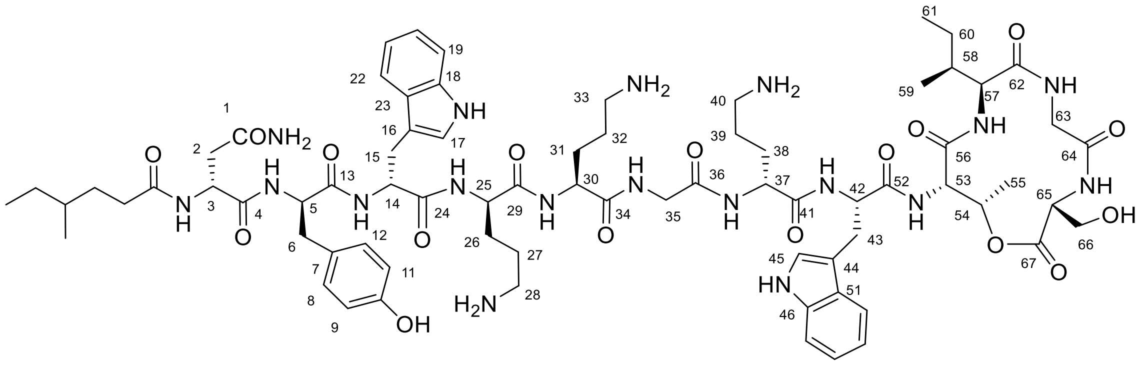

Structure of brevicidine (**1**) with the NMR assignments.

Supplementary Table 3 NMR data of brevicidine A (d_6-DMSO).

position	^{13}CNMR	^{1}HNMR	position	^{13}CNMR	^{1}HNMR
1	172.0		37-NH		8.11 (d, 6.5)
2	36.9	2.35 (d, 7.0, 15.0), 2.53 m	38	29.5	1.40, m 1.32, m
3	49.6	4.54, m	39	23.3	1.31, m
3-NH		8.02 (d, 8.0)	40	38.4	2.59, m
4	172.0		40-NH2		7.65 (2H, brs)
5	54.8	4.27, m	41	171.3	
5-NH		7.99 (d, 7.5)	42	52.9	4.73 (dt, 7.5, 6.5)
6	36.1	2.61 (dd, 8.0, 14.0), 2.79 m	42-NH		8.19 (d, 7.5)
7	127.6		43	27.8	3.08 (dd, 7.5, 15.0) 2.96 (dd, 7.5, 15.0)
8, 12	130.0	6.85 (2H, d, 8.2)	44	109.6	
9, 11	114.9	6.55 (2H, d, 8.2)	45	124.0	7.10 brs
10	155.8		46	136.1	
13	171.2		47	111.4	7.33 (d, 8.0)
14	53.9	4.49, m	48	120.8	7.04 (t, 8.0)
14-NH		8.13 (d, 6.5)	49	118.3	6.98 (t, 8.0)
15	27.2	3.17 m, 3.03 (dd, 10.0, 14.5)	50	118.4	7.57 (d, 8.0)
16	110.1		51	127.4	
17	123.7	7.17 brs	52	170.9	
18	136.1		53	53.9	4.55, m
19	111.3	7.31 (d, 7.9)	53-NH		8.21 (d, 7.5)
20	120.9	7.07 (t, 7.9)	54	69.8	4.90, m
21	118.2	6.95 (t, 7.9)	55	14.0	1.09 (d, 6.5)
22	118.4	7.57 (d, 7.9)	56	167.0	
23	127.3		57	58.8	4.12 (dd, 9.5, 10.5)
24	171.3		57-NH		8.53 (d, 9.5)
25	51.4	4.35, m	58	34.4	1.84, m
25-NH		8.06 (d, 7.5)	59	15.4	0.84 (d, 7.0)
26	29.5	1.76, m 1.53, m	60	24.5	1.05 m, 1.51, m
27	23.6	1.54 (2H, m)	61	10.5	0.85 (t, 7.5)
28	38.5	2.78 (2H, m)	62	171.1	
28-NH2		7.73 (2H, brs)	63	43.3	3.51 (dd, 6.0, 15.0), 3.90 (dd, 6.5, 15.0)
29	171.5		64	169.1	
30	51.8	4.37, m	64-NH		7.6 (d, 8.6)
30-NH		7.92 (d, 7.0)	65	54.4	4.43 (dd, 4.5, 8.0)
31	29.5	1.76, m 1.53, m	66	61.3	3.60(dd, 4.5, 12.0),3.76 (dd, 7.0, 12.0)
32	23.5	1.54 (2H, m)	67	168.9	
33	38.5	2.78 (2H, m)	F-1	172.7	
33-NH2		7.73 (2H, brs)	F-2	33.0	2.07 t
34	171.4		F-3	31.8	1.26, m 1.50, m
35	41.7	3.74, 3.83	F-4	33.5	1.27,m
35-NH		8.23 (d, 6.5)	F-5	29.1	1.09, m 1.27, m
36	168.4		F-6	11.2	0.81 (t,7.5)
37	51.8	4.38, m	F-7	18.8	0.81 (d, 6.5)

^{1}HNMR of brevicidine **(1)** (DMSO-d_6, 500MHz)

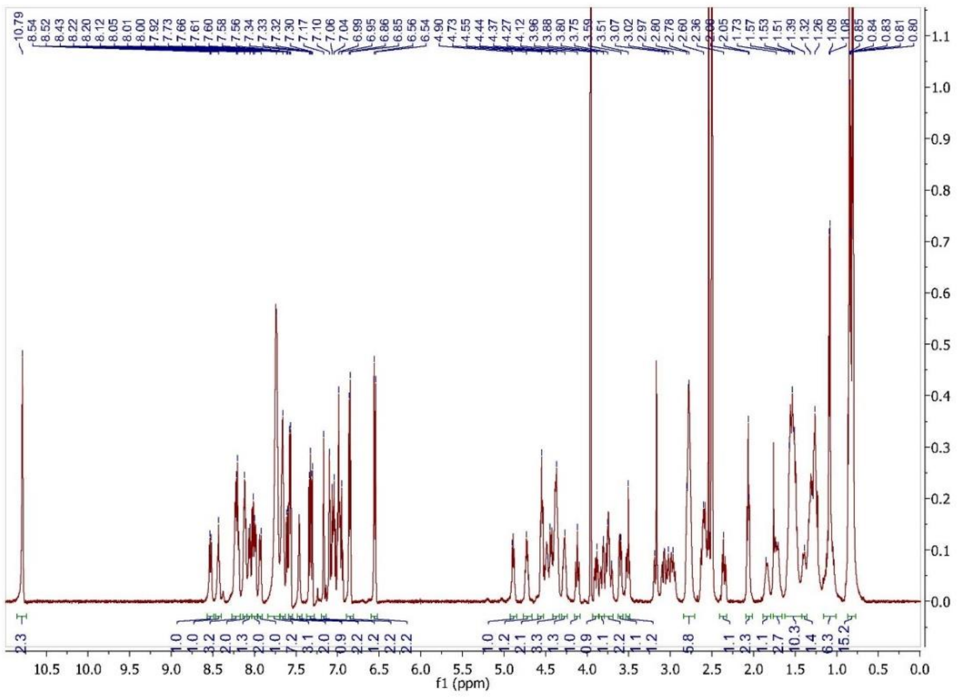

13C-NMR of brevicidine **(1)** (DMSO-d_6, 100MHz)

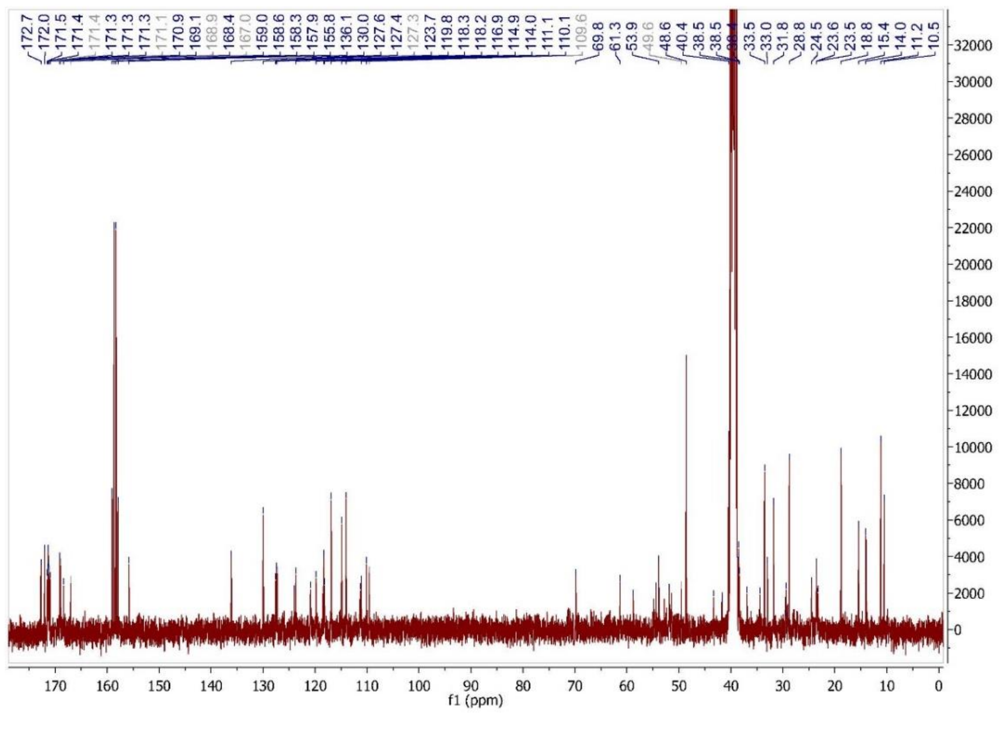

^1H-^1H COSY of brevicidine **(1)** (DMSO-d_6, 500MHz)

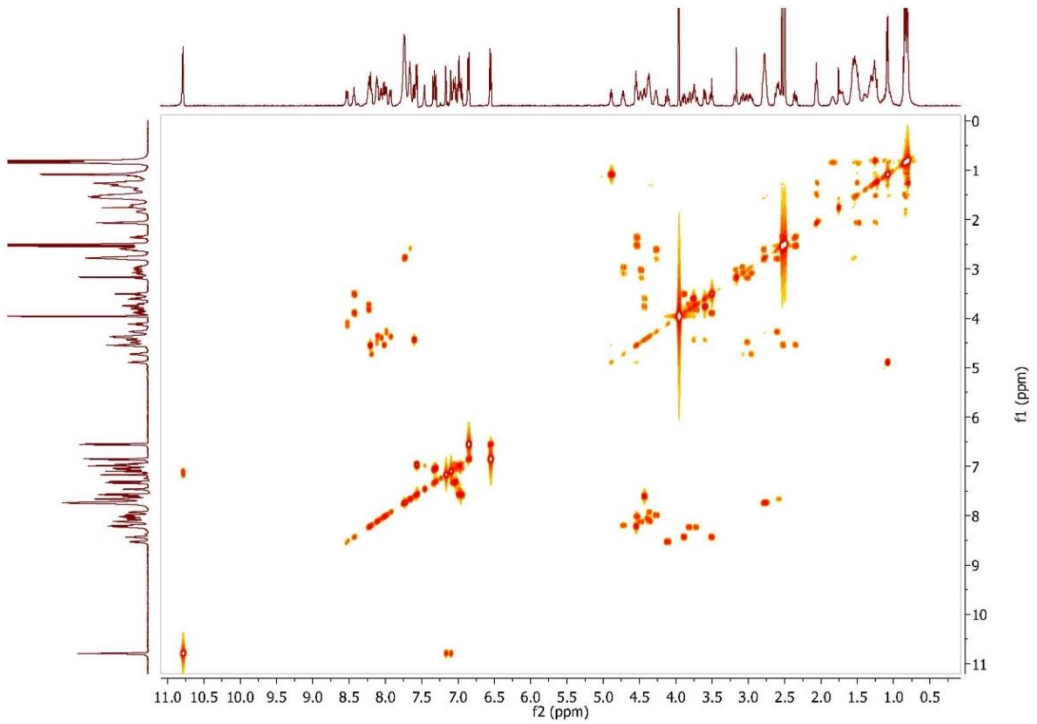

^1H -^{13}C HSQC of brevicidine **(1)** (DMSO-d_6, 500MHz)

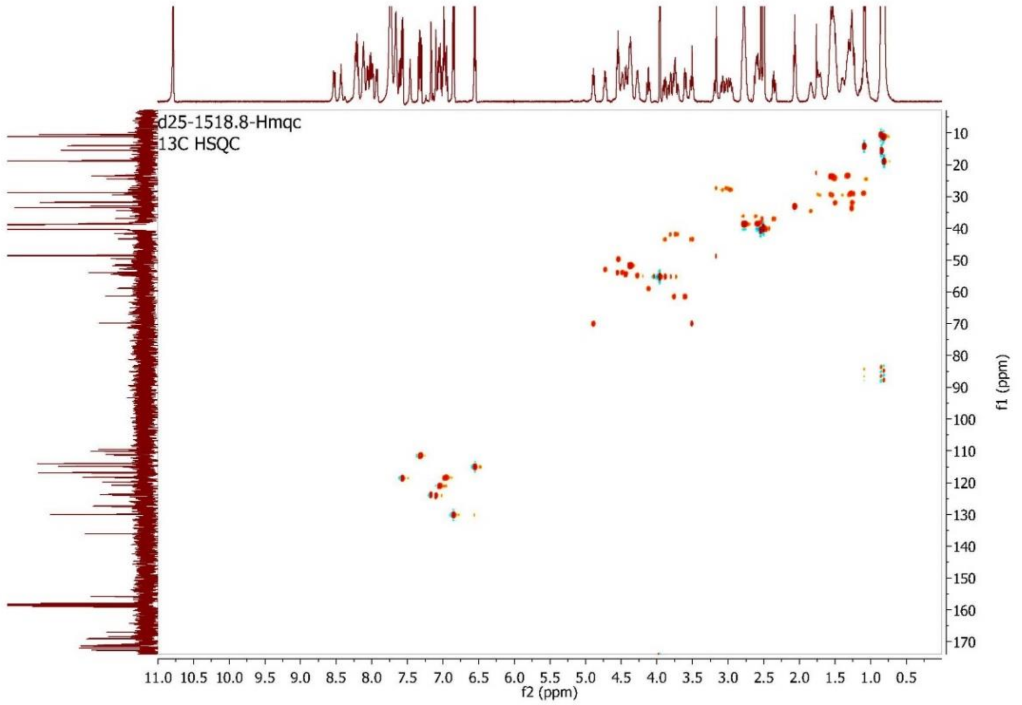

^{1}H -^{13}C HMBC of brevicidine **(1)** (DMSO-d_6, 500MHz)

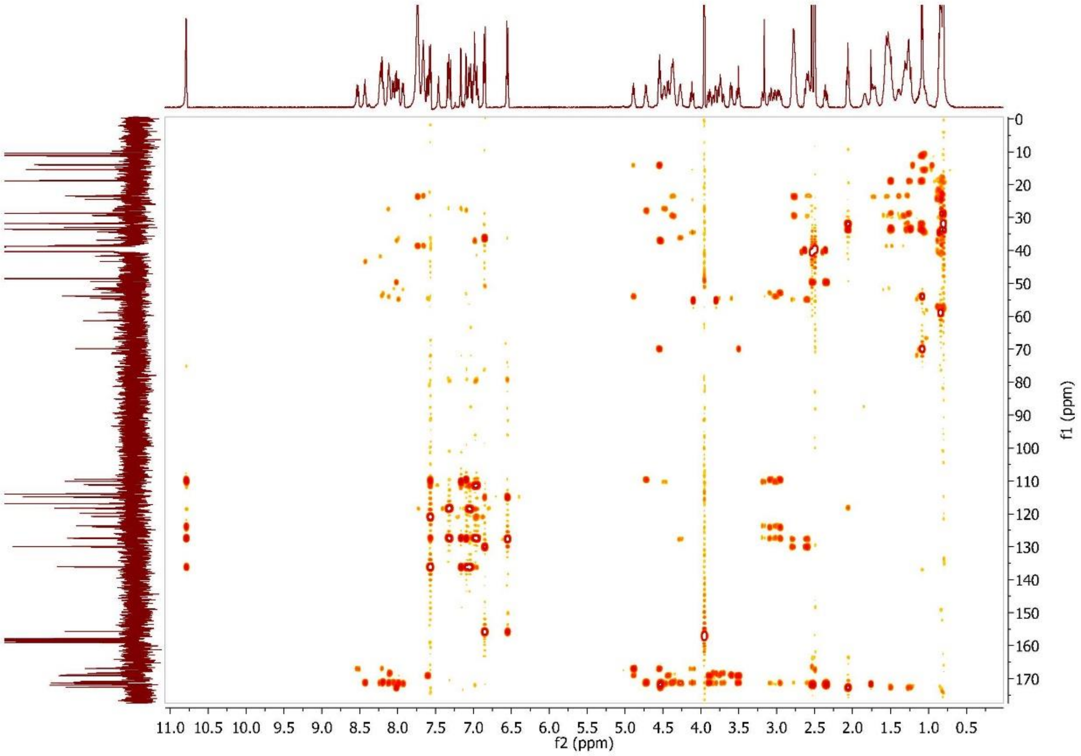

^{1}H-^{1}H TOCSY of brevicidine **(1)** (DMSO-d_6, 500MHz)

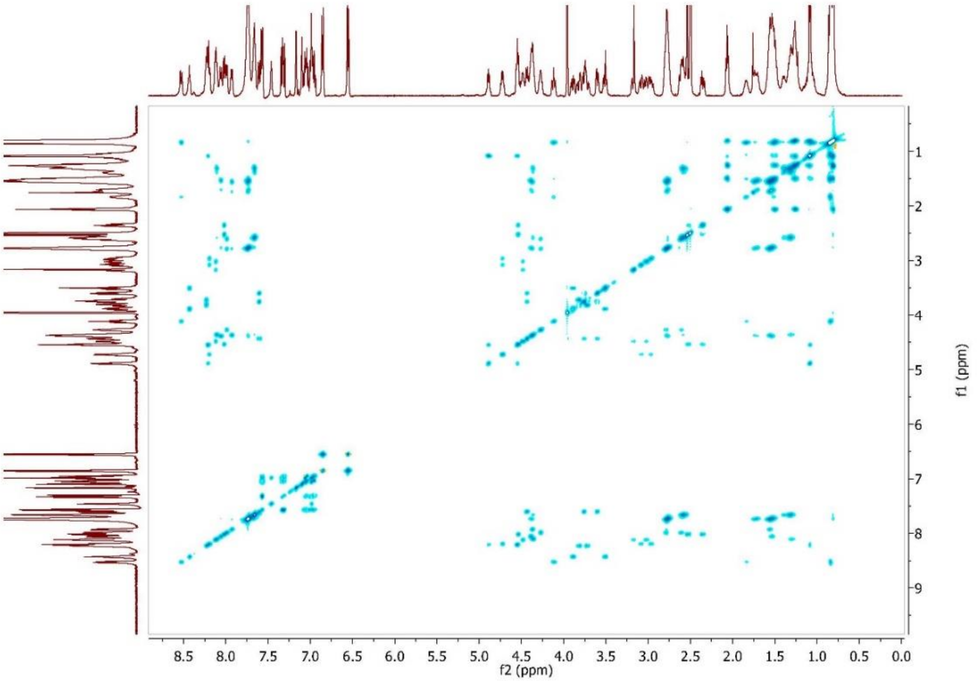

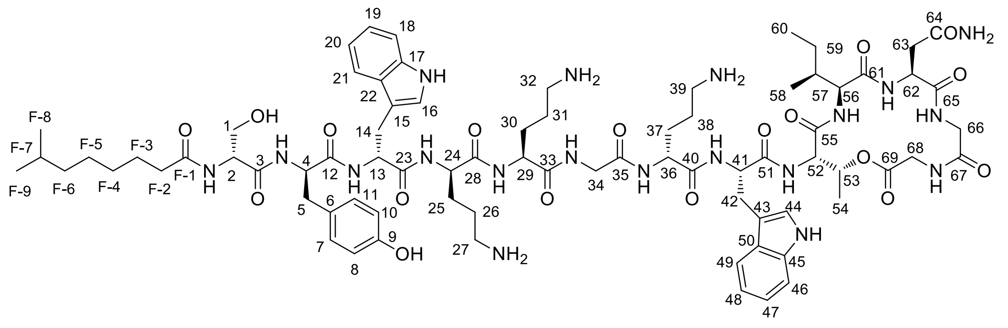

Structure of laterocidine (**2**) with the NMR assignments.

Supplementary Table 4 NMR data of laterocidine (d_6-DMSO).

position	^{13}CNMR	^{1}HNMR	position	^{13}CNMR	^{1}HNMR
1	61.3	3.50 (2H, m)	39	38.1	2.58 (2H, m)
1-OH		5.13, brs	39-NH2		7.65, m
2	54.7	4.26 (dt, 6.5, 7.0)	40	171.1	
2-NH		7.85 (d, 7.5)	41	52.7	4.75 (dt, 5.5, 8.5)
3	170.7		41-NH		8.33, s
4	54.3	4.33, m	42	27.1	2.94, m 3.14, m
4-NH		8.01	43	109.8	
5	35.8	2.62, m, 2.82 (dd, 4.0, 14.0)	44	123.5	7.14 (d, 8.5)
6	127.6		44-NH		10.77 (d, 15.0)
7	129.8	6.89 (d, 8.0)	45	136	
8	114.7	6.56 (d, 8.0)	46	111.1	7.32 (t, 8.5)
9	155.8		47	120.7	7.05, m
9-OH		9.16	48	118	6.97, m
10	114.7	6.56 (d, 8.0)	49	118.2	7.57 (d, 8.0)
11	129.8	6.89 (d, 8.0)	50	127.2	
12	171.2		51	171.8	
13	53.3	4.51 (dt, 5.0, 8.0)	52	53.7	4.68 (dd, 3.0, 9.0)
13-NH		8.0, m	52-NH		7.92 (d, 9.0)
14	27.1	2.94, m 3.14, m	53	71.7	5.17, m
15	109.8		54	15.2	1.09 (3H, d ,6.5)
16	123.5	7.14 (d, 8.5)	55	168.7	
16-NH		10.77 (d, 15.0)	56	58.1	3.90, m
17	136		56-NH		8.30, m
18	111.1	7.32 (t, 8.5)	57	35.5	1.57, m
19	120.7	7.05, m	58	14.6	0.85 (3H, d ,6.5)
20	118	6.97, m	59	24.9	1.15, m 1.55, m
21	118.2	7.57 (d, 8.0)	60	10.8	0.87 (3H, t, 7.5)
22	127.2		61	173.2	
23	171.5		62	51.2	4.12, m

24	51.2	4.41, m	62-NH		9.40 (d,6.5)
24-NH		8.09 (d,8.0)	63	34.9	2.66, m 2.88 (dd, 3.5, 16.5)
25	29.1	1.51, m 1.70, m	64	171.9	
26	23.2	1.51 (2H, m)	64-NH2		7.74
27	38.2	2.75 (2H, m)	65	172.8	
27-NH2		7.76, brs	66	42.4	3.58 (dd, 17.0, 4.0) 4.02 (dd, 17.0, 8.0)
28	171.2		66-NH		8.03
29	51.2	4.39, m	67	169.4	
29-NH		8.02	68	40.8	3.38, m 4.45 (dd, 10.0, 6.5)
30	29.1	1.54, m 1.74, m	68-NH		7.68
31	23.2	1.58 (2H, m)	69	168.9	
32	38.2	2.79 (2H, m)	F-1	172.7	
32-NH2		7.74, brs	F-2	34.8	2.11 (2H, m)
33	171.4		F-3	25	1.45 (2H, m)
34	41.5	3.75 (dd, 17.5, 4.0), 3.85	F-4	28.7	1.20 (2H, m)
34-NH		8.24, m	F-5	28.7	1.23 (2H, m)
35	168.3		F-6	38.1	1.11 (2H, m)
36	51.2	4.38, m	F-7	27.2	1.47, m
36-NH		8.06 (d, 8.0)	F-8	22.3	0.83 (3H, d,6.5)
37	29.2	1.28, m 1.43, m	F-9	22.3	0.83 (3H, d,6.5)
38	22.9	1.29 (2H, m)			

^{1}HNMR of laterocidine **(2)** (DMSO-d_6, 500MHz)

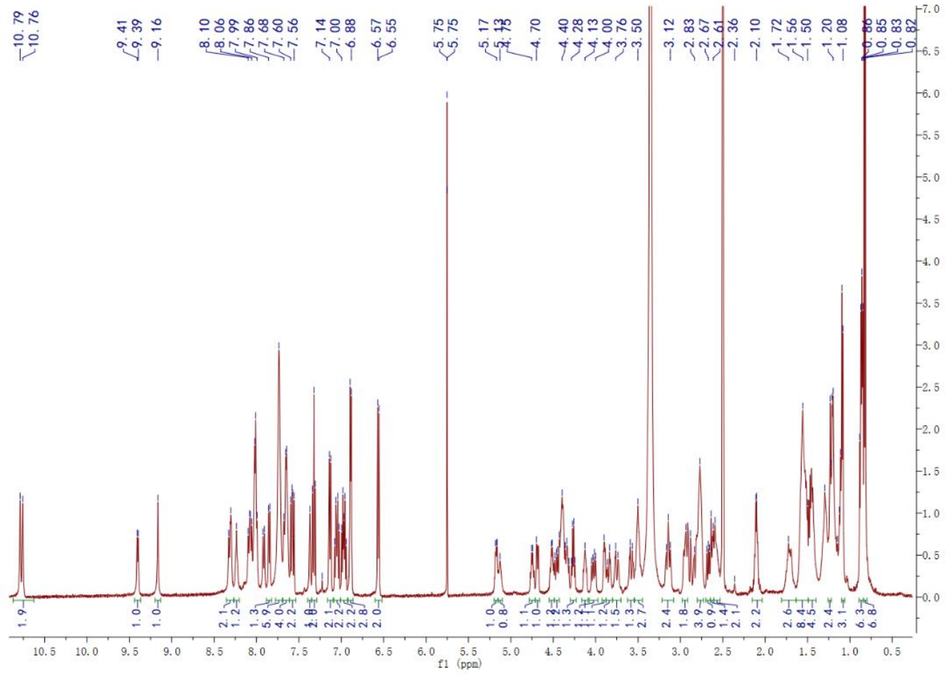

^{1}H-^{1}H COSY of laterocidine **(2)** (DMSO-d_6, 500MHz)

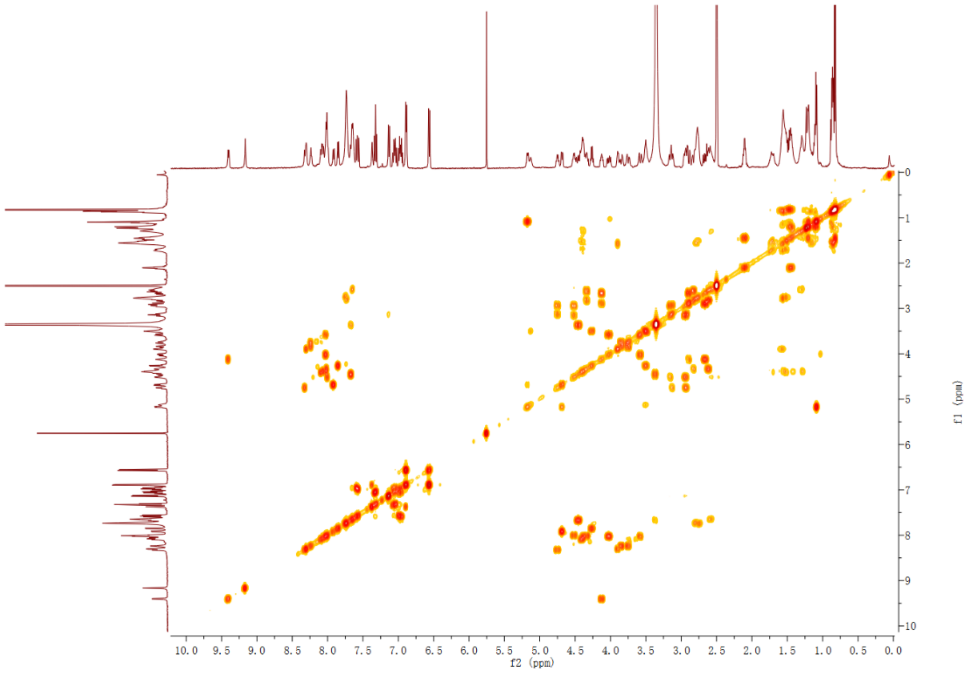

^{1}H -^{13}C HSQC of laterocidine **(2)** (DMSO-d_6, 500MHz)

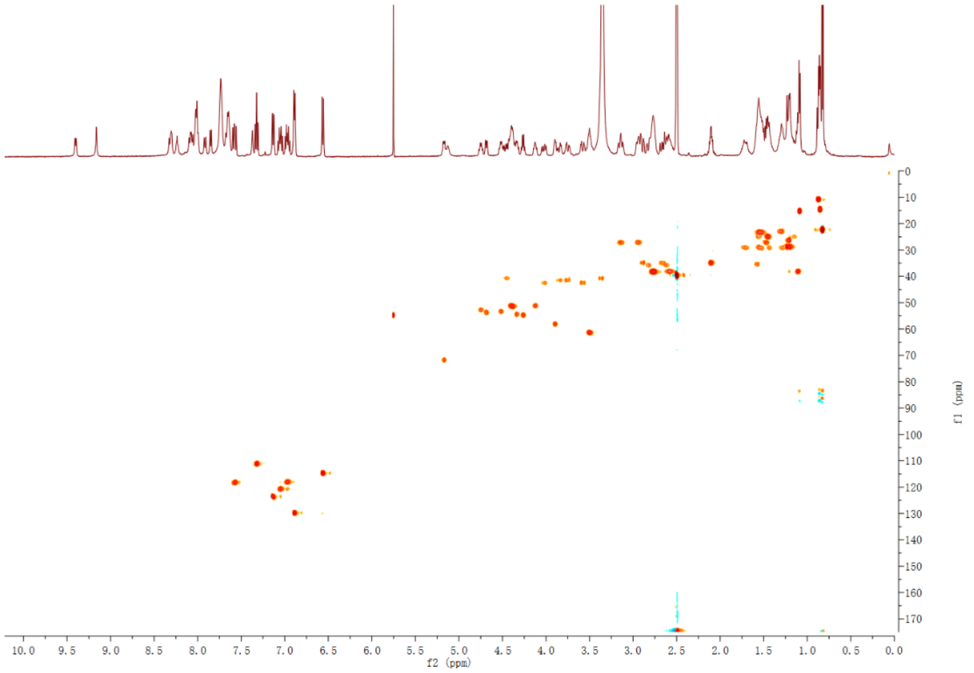

^{1}H -^{13}C HMBC of laterocidine **(2)** (DMSO-d_6, 500MHz)

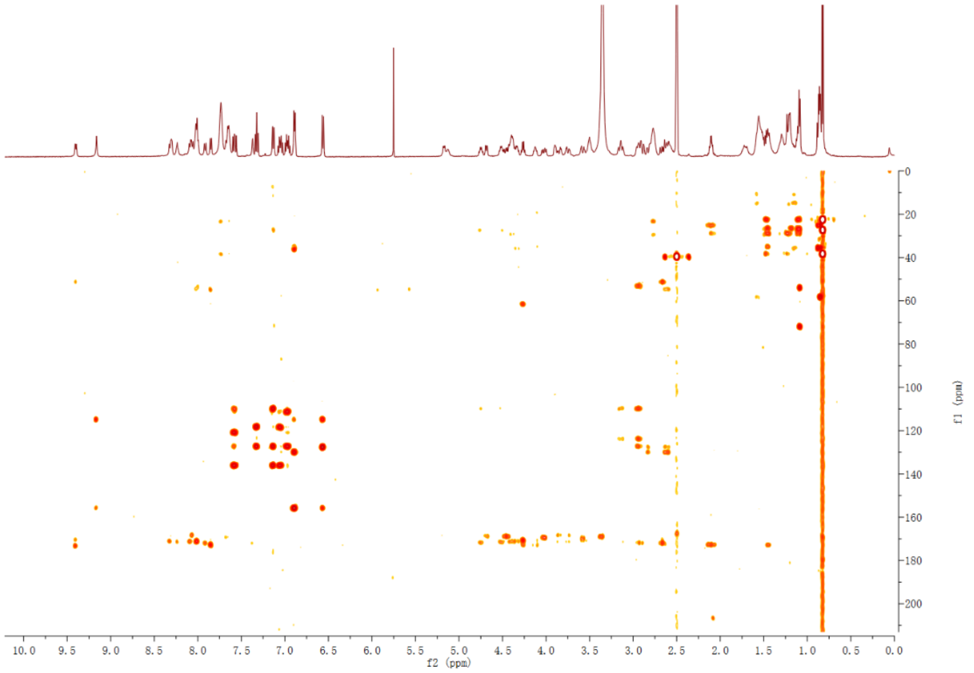

^{1}H-^{1}H TOCSY of laterocidine **(2)** (DMSO-d_6, 500MHz)

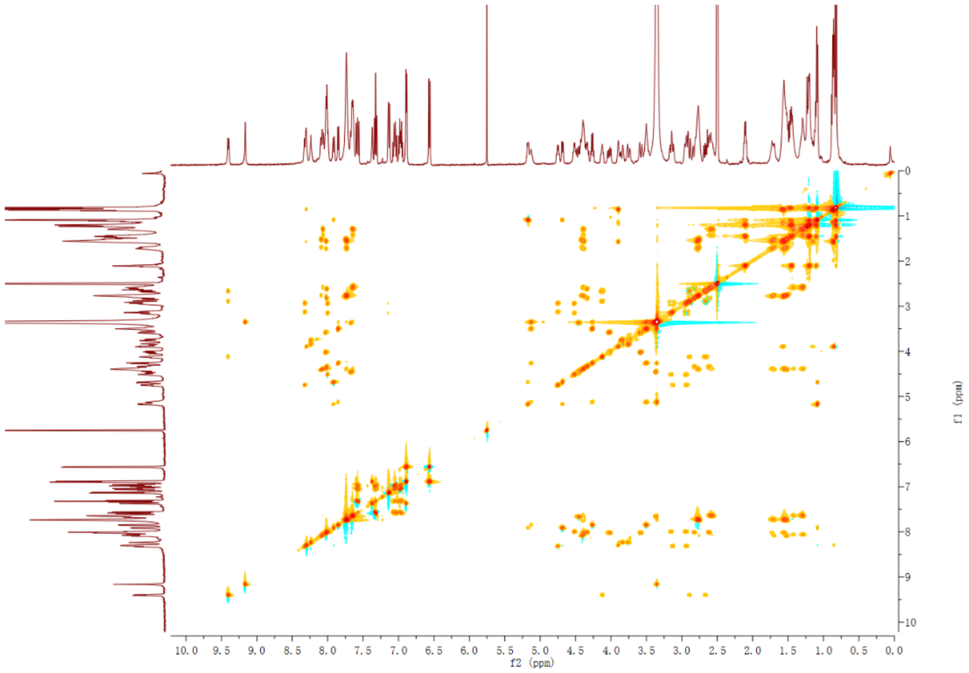

f2 (ppm)
f1 (ppm)

NMR data of compound 4.

Supplementary Table 5. NMR data of **4** (CD_3OD).

position	^{13}C	^{1}H	position	^{13}C	^{1}H
1	54.8	4.26 (t, 7.5)	14	14.7	1.25 (d, 6.4, 3H)
2	28.9	3.24 (dd, 7,5, 15.0) 3.39 (dd, 7,5,	15	169.3	
3	107.8		16	59.9	4.22 (dd, 9.5, 10.5)
4	125.9	7.21 brs	16-NH		8.60 (d, 9.6)
5	138.5		17	35.8	1.94, m
6	112.7	7.38 (d, 8.0)	18	15.8	0.93 (d, 6.7)
7	122.9	7.13 (t, 8.0)	19	24.9	1.16 m, 1.62, m
8	120.4	7.06 (t, 8.0)	20	11.2	0.95 (t, 7.4)
9	118.4	7.62 (d, 8.0)	21	174.7	
10	128.4		22	44.7	3.37 (d, 14.7), 4.37 (d, 14.7)
11	170.1		23	171.9	
12	55.9	4.67, (d, 5.1)	24	54.4	4.50 (dd, 4.9, 3.7)
13	71.9	5.22, (q, 6.4)	25	62.9	3.97 (dd, 3.6, 11.0),3.82 (dd, 4.0, 11.0)
14	14.7	1.25 (d, 6.4, 3H)	26	170.4	

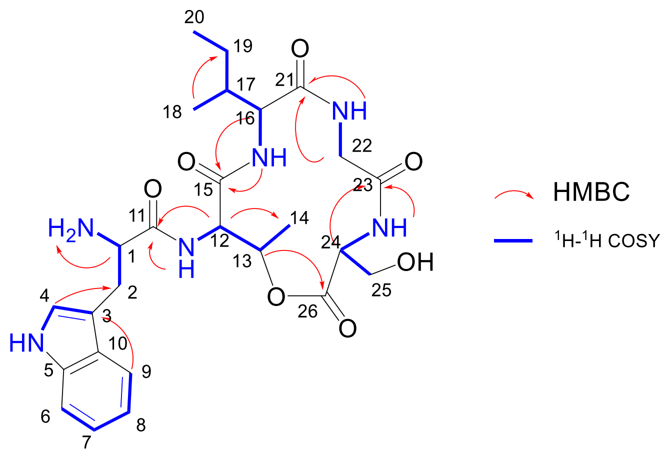

Key ^{1}H-^{1}H COSY and HMBC correlation of **4**

^{1}HNMR of **4** (CD_3OD, 500MHz)

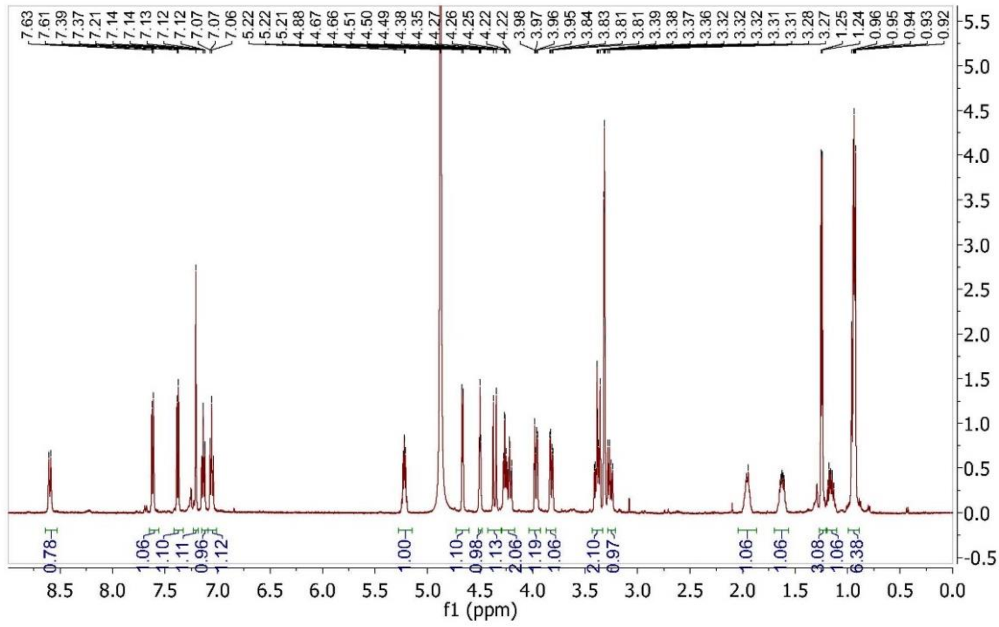

^{13}NMR of **4** (CD_3OD, 100MHz)

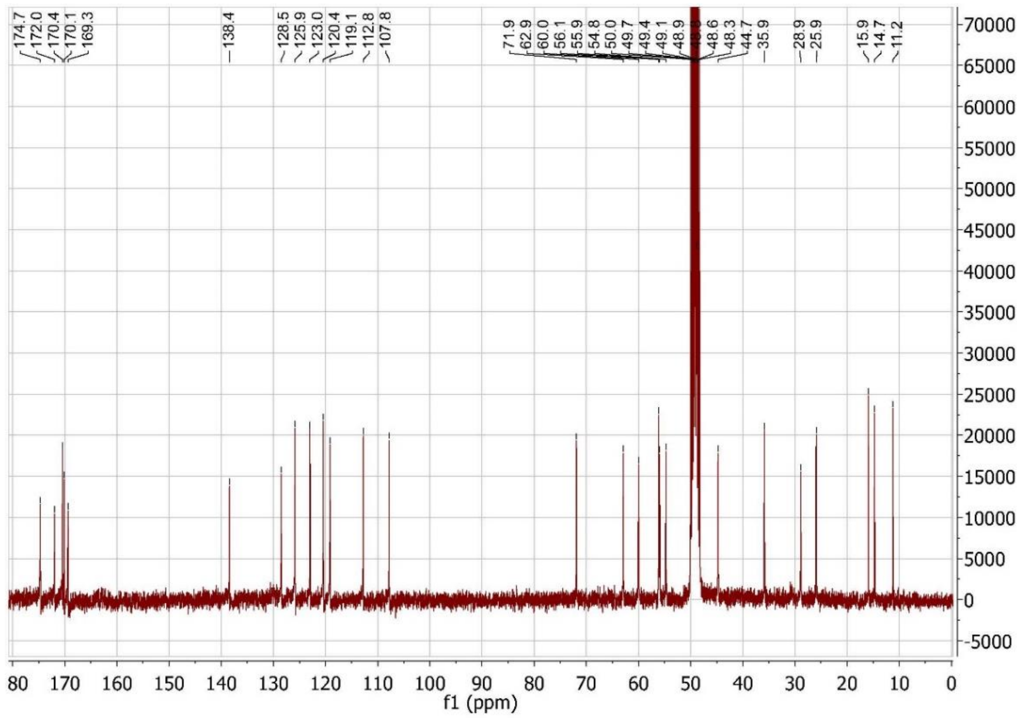

^{1}H-^{1}H COSY of **4** (CD_3OD, 500MHz)

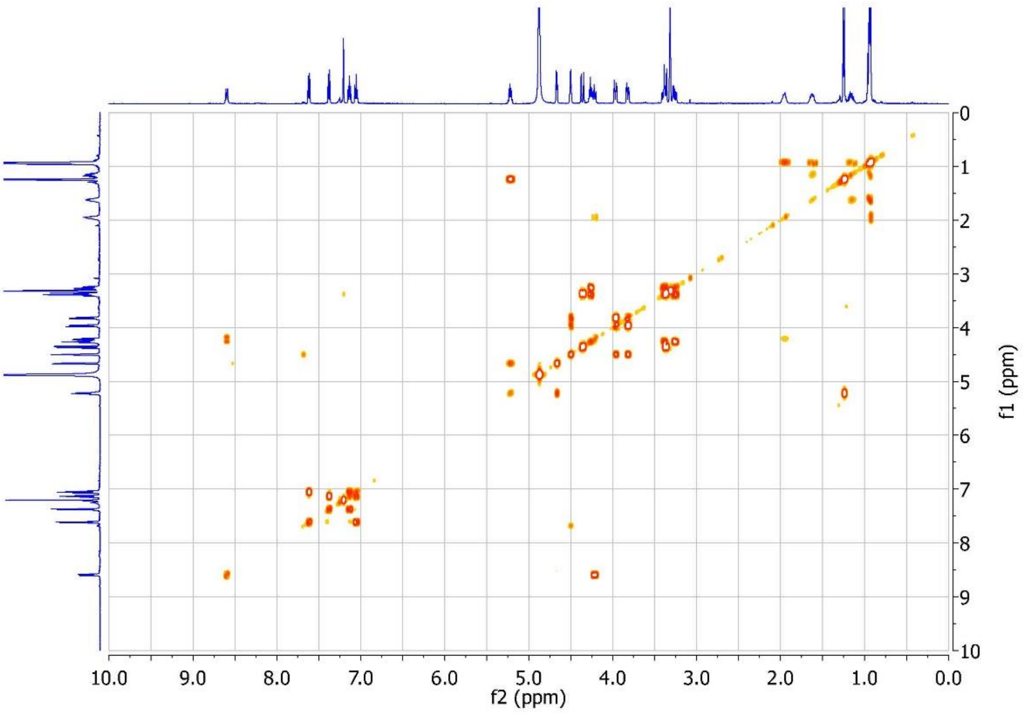

^{1}H -^{13}C HSQC of **4** (CD_3OD, 500MHz)

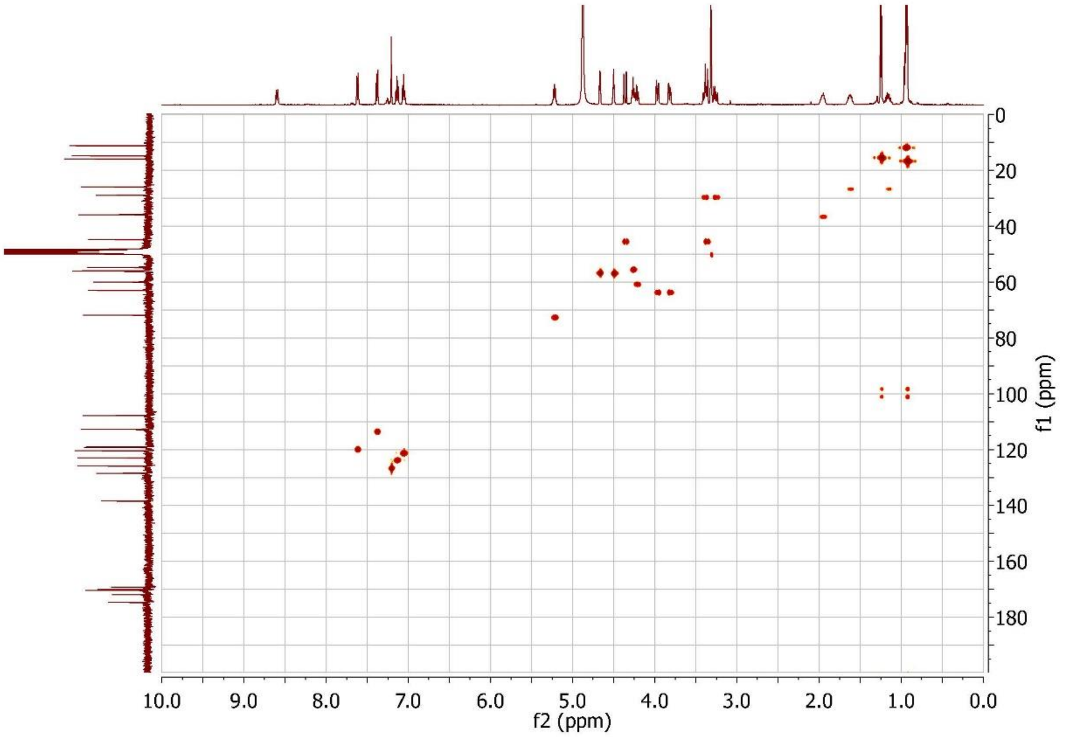

^{1}H - ^{13}C HMBC of **4** (CD_3OD, 500MHz)

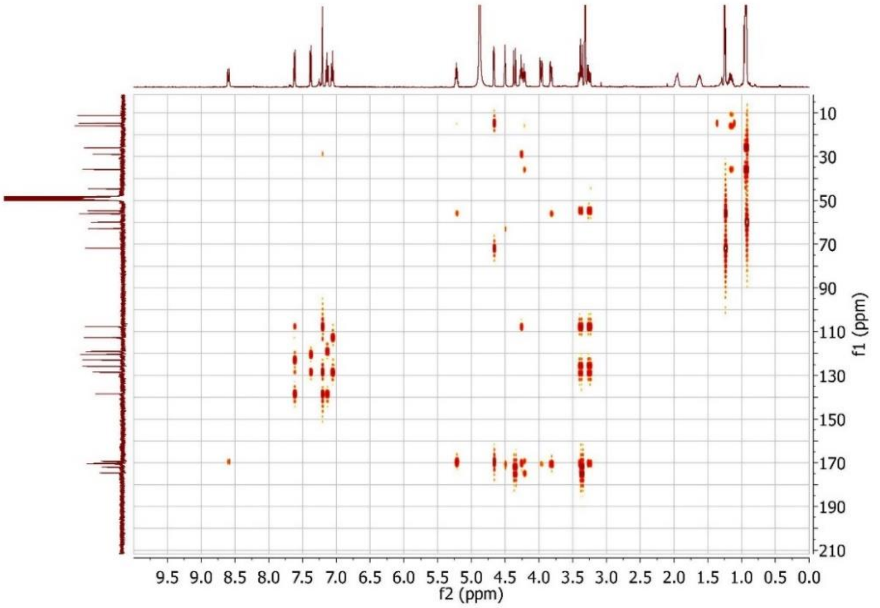

Compound characterization by MS analysis

Structure	Compound	MSn fragments
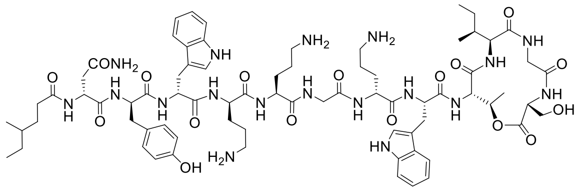	**1**	$[M+2H]^{2+}$ 760.4<br>y: 359.3, 545.2, 659.3, 716.3, 830.4, 944.4, 1130.5, 1293.5, 1389.6(-H_2O)<br>b: 227.1, 390.3, 576.3, 690.3, 804.4, 861.4, 975.4, 1161.5, 1244.5(-H_2O)
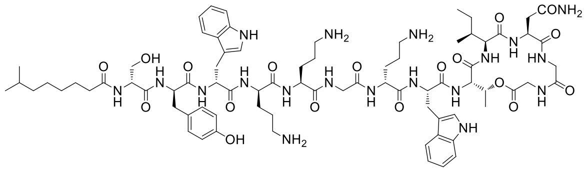	**2**	$[M+2H]^{2+}$ 802.9<br>y: 443.3, 629.3, 914.4, 1028.5, 1214.5<br>b: 391.3, 577.3, 691.4, 805.4, 976.5, 1162.5
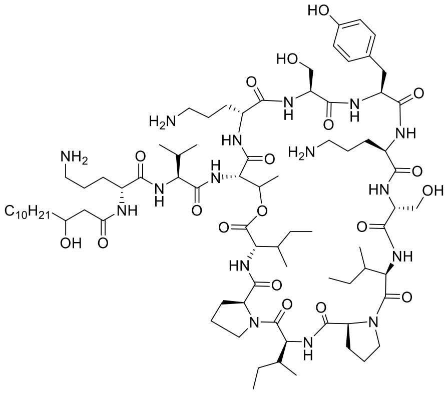	**3**	$[M+2H]^{2+}$ 813.5
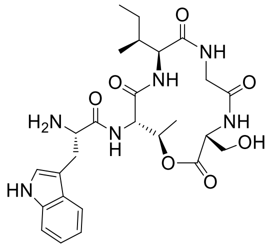	**4**	$[M+H]^{+}$ 545.3<br>y: 276.2, 359.2<br>b: 458.3
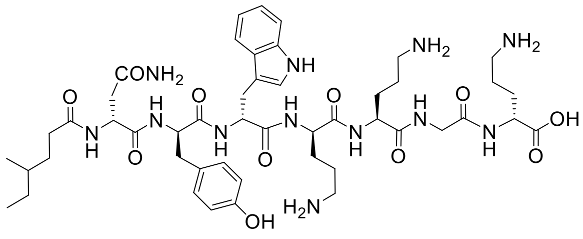	**5**	$[M+H]^{+}$ 992.54<br>y: 190.1, 304.2, 418.3, 604.4, 767.4, 881.5<br>b: 227.1, 390.2, 576.3, 690.4, 804.5, 861.5

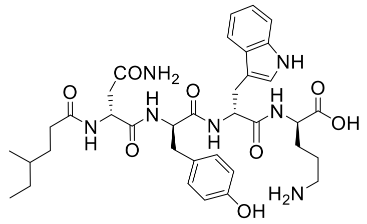	**6**	$[M+H]^+$ 707.36<br>y: 319.2, 482.3, 596.3<br>b: 390.2, 576.3
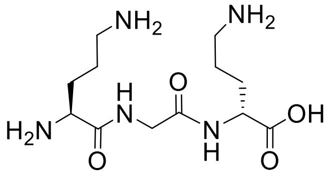	**7**	$[M+H]^+$ 304.2<br>y: 133.1, 190.1<br>b: 115.1, 172.1
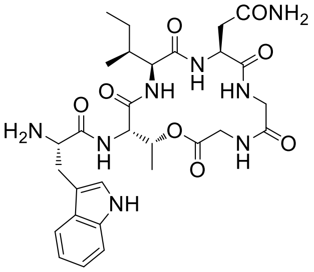	**8**	$[M+H]^+$ 629.3<br>b: 515.3, 553.2
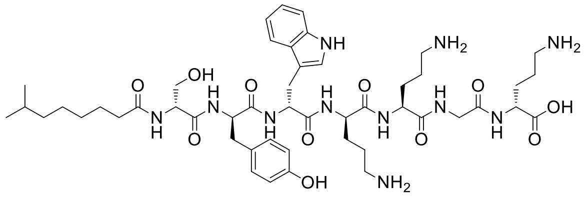	**9**	$[M+2H]^{2+}$ 497.3<br>y: 190.1, 304.2, 418.3, 604.4, 767.4, 854.4<br>b: 228.2, 391.3, 577.3, 691.4, 805.4
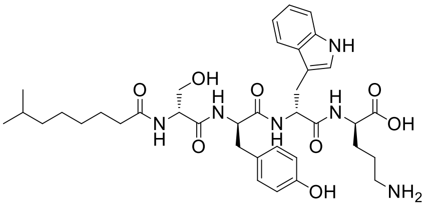	**10**	$[M+H]^+$ 709.4<br>y: 319.2, 482.2, 569.2<br>b: 391.2, 577.2
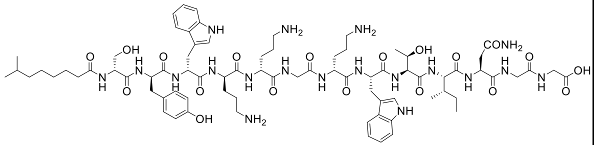	**11**	$[M+2H]2^+$ 811.9<br>y: 247.1, 360.2, 461.2, 761.4, 818.4, 932.4, 1046.5, 1232.5<br>b: 391.3, 577.3, 691.4, 862.4, 976.5, 1162.5, 1263.5, 1376.6
