# Supplementary material for: Discovery of cationic nonribosomal peptides as Gram-negative antibiotics through global genome mining
Source: Nat Commun. 2018 Aug 16;9:3273. doi: 10.1038/s41467-018-05781-6 (PMC6095874; doi:10.1038/s41467-018-05781-6)
Supplement: Supplementary file 2 — Description of Additional Supplementary Files [file 41467_2018_5781_MOESM2_ESM.pdf]

Description of Additional Supplementary Files

File Name: Supplementary Data 1
Description: To gain insight into the biosynthetic capacity of bacterial CNRPs, we created a series of scripts to collect BGC information from the antiSMASH annotated files. Costume Node.js scripts were prepared to extract predicted amino acid building blocks from adenylation (A) domains in NRPS gene clusters.

counterRelease.js: creates json file containing predicted amino acid building blocks from adenylation (A) domains in NRPS gene clusters in all antiSMASH results files.

release.js: creates result tabular txt files, require json file created by counterRelease.js.

others: core scripts and linking scripts for extraction of gene clusters' information (domains, predicted amino acids from A domain, SMCOG etc.).
